# Cryo-EM structure of human Cx31.3/GJC3 connexin hemichannel

**DOI:** 10.1126/sciadv.aba4996

**Published:** 2020-08-28

**Authors:** Hyuk-Joon Lee, Hyeongseop Jeong, Jaekyung Hyun, Bumhan Ryu, Kunwoong Park, Hyun-Ho Lim, Jejoong Yoo, Jae-Sung Woo

**Affiliations:** 1 Department of Life Sciences, Korea University, Seoul 02841, Republic of Korea.; 2 Korea Basic Science Institute, Chungcheongbuk-do 28119, Republic of Korea.; 3 Molecular Cryo-electron Microscopy Unit, Okinawa Institute of Science and Technology Graduate University, Okinawa 904-0496, Japan.; 4 Neurovascular Unit Research Group, Korea Brain Research Institute (KBRI), 41062 Daegu, Republic of Korea.; 5 Department of Physics, Sungkyunkwan University, Suwon, Republic of Korea.; 6 Center for Self-assembly and Complexity (CSC), Institute for Basic Science (IBS), Pohang 37673, Republic of Korea.

## Abstract

Connexin family proteins assemble into hexameric channels called hemichannels/connexons, which function as transmembrane channels or dock together to form gap junction intercellular channels (GJIChs). We determined the cryo–electron microscopy structures of human connexin 31.3 (Cx31.3)/GJC3 hemichannels in the presence and absence of calcium ions and with a hearing-loss mutation R15G at 2.3-, 2.5-, and 2.6-Å resolutions, respectively. Compared with available structures of GJICh in open conformation, Cx31.3 hemichannel shows substantial structural changes of highly conserved regions in the connexin family, including opening of calcium ion–binding tunnels, reorganization of salt-bridge networks, exposure of lipid-binding sites, and collocation of amino-terminal helices at the cytoplasmic entrance. We also found that the hemichannel has a pore with a diameter of ~8 Å and selectively transports chloride ions. Our study provides structural insights into the permeant selectivity of Cx31.3 hemichannel.

## Teaser

Cryo-EM structure of human connexin 31.3 hemichannel reveals the selective permeability mechanism.

## INTRODUCTION

Gap junction facilitates direct communication between neighboring cells and plays key roles in numerous cellular processes such as cardiac contraction, ionic transmission in electrical synapse, development, and differentiation. In vertebrates, various connexin family proteins assemble into hexameric channels called hemichannels/connexons in cell membrane, and two hemichannels from adjacent cells dock together to form a gap junction intercellular channel (GJICh) (*1*–*3*). While GJIChs provide passages for ions and metabolites to freely diffuse through two adjacent cell membranes (*4*, *5*), hemichannels participate in signal transduction through the release of small molecules such as adenosine 5′-triphosphate (ATP), nicotinamide adenine dinucleotide, and glutamate (*6*–*9*). The gating and permeability of both hemichannels and gap junction channels are directly regulated by various factors such as transjunctional/transmembrane voltage, pH, divalent ions, and membrane lipids (*10*–*13*).

Twenty human connexin genes, except the highly diversified connexin 23 (Cx23)/GJE1 member, share 55 to 82% amino acid sequence similarity in the regions spanning the N-terminal helix (NTH), four transmembrane helices (TM helices), and two extracellular loops (ECLs) (fig. S1). Because of the sequence similarity, hemichannels and GJIChs are thought to have a common mechanism of gating in response to general regulatory signals. A total of 11 electrically charged residues are identically or similarly conserved in all 20 human connexins (fig. S1), suggesting that these residues may play key roles in channel gating.

Mutations on connexin proteins have been found to cause at least 28 human diseases, including oculodentodigital dysplasia, Charcot-Marie-Tooth neuropathy, keratitis-ichthyosis-deafness (KID) syndrome, cataract, deafness, and leukodystrophy (*14*). In particular, a growing number of evidence shows that connexin mutations impairing hemichannel activity lead to the pathogenicity (*15*). For example, mutated Cx26 and wild-type Cx43 assemble into hyperactive heteromeric hemichannels with increased Ca^2+^ permeability and ATP release, which results in KID syndrome (*16*). Therefore, understanding the detailed gating and permeability regulation mechanisms of hemichannels and GJIChs is of biomedical importance.

Previous studies have suggested that channel gating may involve marked conformational changes of NTHs in connexin protomers (*17*, *18*). Charged residues in NTHs have been believed to form at least part of transjunctional voltage sensor (*19*, *20*). In available high-resolution structures of Cx26 (*21*) and Cx46/50 (*22*) GJIChs, which are thought to represent the open state, NTHs line the inner surface of the channel pore through the interaction with the first and the second TM helices (TM1 and TM2). When this interaction is disturbed by M34A mutation in Cx26, NTHs appeared to interact with each other to form a plug inside of the pore, hence closing the channel (*17*). However, it is not clear whether such plug formation represents a general channel-closing mechanism that is physiologically relevant.

While gradual inhibition of channel activity by increasing the concentration of Ca^2+^ ions has been demonstrated for various hemichannels and GJIChs (*11*, *23*–*25*), the underlying mechanism is still unclear. The crystal structure of Cx26 GJICh in the presence of Ca^2+^ ions has shown that Ca^2+^ ions bind to E42 and E47 residues on the surface of the channel pore, generating a positive electrostatic barrier that may affect permeability and selectivity of transported molecules (*26*). However, since E42 is not conserved in connexin family proteins, this seems to be not a general mechanism of Ca^2+^-dependent closing. In a more recent electrophysiological study on Cx26 hemichannel, D50 and E47 were found to be major Ca^2+^-binding sites (*13*), suggesting that the Ca^2+^-dependent closing mechanism of hemichannel is different from that of GJICh. Clear explanation of the causal relationship between the Ca^2+^ binding to these residues and Cx26 channel closing is precluded because of the lack of high-resolution structure.

Human Cx31.3/GJC3, which is expressed in oligodendrocytes of the central nervous systems, does not form intercellular channels (*9*, *27*). Consistent with the experimental data, some of the residues important for connexon-connexon docking in Cx26 or Cx46/50 GJIChs are not conserved in Cx31.3, suggesting that this connexin functions exclusively as hemichannel. A recent study using immunocytochemistry and electron microscopy (EM) has shown that axolemmal K_V_1.1 channels are precisely aligned with Cx29 (the rodent ortholog of Cx31.3) hemichannels in the surrounding juxtaparanodal myelin collars (*28*), suggesting that structurally coupled K_V_1 and Cx29 channels may facilitate faster axonal repolarizations and/or saltatory conduction of mammalian myelinated axons. Two mutations R15G and L23H in human Cx31.3 have been found to cause nonsyndromic hearing loss, and biochemical study in HeLa cells demonstrated that these mutations inhibit ATP release through Cx31.3 hemichannel (*29*).

In this study, we used single-particle cryo-EM to determine three high-resolution structures of Cx31.3 hemichannels in the presence and absence of Ca^2+^ ions and the R15G-mutant hemichannel, respectively. These structures show the NTH conformation distinct from the previous GJICh structures, putative lipid-binding sites in the inner surface of the pore, and Ca^2+^-binding tunnels in the interprotomer interface and between transmembrane and extracellular regions. Our structural study, together with molecular dynamics (MD) simulations and electrophysiological experiments, reveals the selective permeability mechanism of Cx31.3 hemichannel.

## RESULT

### The overall structure of Cx31.3 hemichannel shows entrance-covering conformation of NTHs

**Fig. 1:**
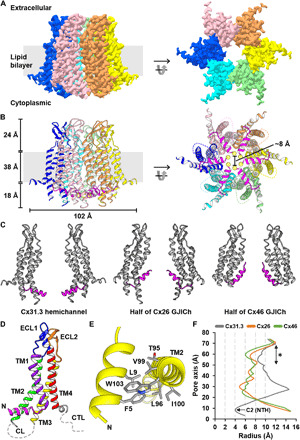
Overall structure of Cx31.3 hemichannel with entrance-covering NTHs. (**A** and **B**) The overall structure of Cx31.3 hemichannel. Single-particle reconstruction map (A) and ribbon representations (B) are shown in the same orientations. Six connexins in the hemichannel are shown in different colors, and NTHs are in magenta color in (B). Yellow spheres indicate sulfur atoms in C2. (**C**) Comparison of NTH conformation in Cx31.3 hemichannel with those of Cx26 and Cx46 GJIChs. Only two interfacing connexins in a hexameric channel are shown for clarity. The accession codes for Cx26 and Cx46 GJICh structures are 2ZW3 and 6MHQ, respectively. NTHs are shown in magenta color. (**D**) Ribbon presentation of a single Cx31.3 connexin. NTH, TM helices, and ECLs are shown in different colors and are labeled. Cytoplasmic loop (CL) and C-terminal loop (CTL) are represented as gray dashed lines. (**E**) Intramolecular hydrophobic interactions between NTH and TM2. (**F**) The solvent-accessible pore radius through Cx31.3 hemichannel pore (gray) is compared with those through Cx26 (orange) and Cx46 (green) GJICh pores. Locations of C2 and the acidic band of Cx31.3 are indicated using an arrow and an asterisk.

To gain insights into the gating and permeability regulation mechanisms of connexin hemichannels and GJIChs, we determined the structure of Cx31.3 hemichannel solubilized in lauryl maltose neopentyl glycol (LMNG) at 2.3 Å using single-particle cryo-EM (Fig. 1A and fig. S2). The overall structure and hexameric interaction of Cx31.3 hemichannel are similar to those of Cx26 and Cx46/50 GJIChs, as expected by high sequence homology between these connexins. However, the conformation of NTHs in hemichannel shows substantial difference from those of GJIChs. Six NTHs in the hemichannel are placed at the cytoplasmic entrance, whereas those in GJIChs are at the inner surface of the pore (Fig. 1, B to D). The entrance-covering conformation appears to be stabilized mainly by intramolecular hydrophobic interactions of F5 and L9 in NTH, with T95, L96, V99, I100, and W103 in TM2 (Fig. 1E). Notably, five of these residues are hydrophobically conserved in A- and C-class connexins, including Cx43 and Cx46 (fig. S1), suggesting that these connexins may also adopt a similar conformation in their hemichannel states. We also found the interprotomer interaction between NTH and TM2, including the hydrophobic interaction between I100 and L10 and the cation-π interaction of R7 with W103 or F5 (fig. S3A). However, these interactions are relatively superficial compared with the intramolecular interaction, and majority of the interacting residues are not conserved in other connexins.

Since six N termini in the entrance-covering NTH conformation face toward the pore center and line the smallest pore at the cytoplasmic entrance (Fig. 1F), N-terminal modifications could play a important role in substrate permeability and selectivity. We performed mass spectrometry analysis of purified Cx31.3 hemichannels and found that the first methionine residue (M1) was completely removed, and the N terminus of the second cysteine residue (C2) was partially acetylated (fig. S3B). In addition, we reconstructed the density map without imposing any symmetry to find possible conformational changes between acetylated and unacetylated N termini. While we identified several different N-terminal conformations, it was not clear whether the weak densities observed in these regions are caused by acetylation or are randomly formed (fig. S3, C and D). Hence, in our structural model, which is built into the density map with C6 symmetry, we included only unacetylated cysteine residues at six N termini in a single major conformation. With this structure, we analyzed the solvent-accessible pore diameters along the pore axis and found that NTHs form the smallest pore with a diameter of ~8 Å, which is smaller than those of Cx26 (10 Å) and Cx46/50 (11 Å) GJICh structures (Fig. 1F).

To confirm whether the Cx31.3 structure determined in a detergent environment is also stable in a lipid bilayer, we used MD simulations. We reconstructed six cytoplasmic loops (CLs; residues 108 to 126) that are missing in the hemichannel structure and reconstituted the resulting structure into a lipid bilayer surrounded by an ionic solution containing 150 mM KCl (see Materials and Methods for details) (fig. S3E). In a 1.5-μs-long MD simulation at 300 K, which is computed excluding six CLs, the root mean square deviation (RMSD) of the channel converged to about 3.5 Å, suggesting that the channel remains structurally stable under a thermal fluctuation (fig. S3F). To see whether there exists particularly dynamic structural motifs, we computed the root mean square fluctuation (RMSF) using the 1.5-μs trajectory (fig. S3G). The result showed the RMSF values of ~1 Å for all residues, except those in six CLs and several terminal residues, suggesting that the hemichannel structure is stable in a lipid bilayer. We also performed MD simulation of a separate connexin protomer in the same lipid and solution environment (fig. S3H), and the RMSD and RMSF measurements of a protomer during a 200-ns trajectory showed quantitatively similar trends to those of a hemichannel (fig. S3, I and J). This result, together with the strong intramolecular interaction between NTH and TM2, suggests that the NTH conformation shown in the hemichannel structure may have already been set in the monomeric state of connexin.

### The putative lipid-binding sites in the pore cavity

**Fig. 2:**
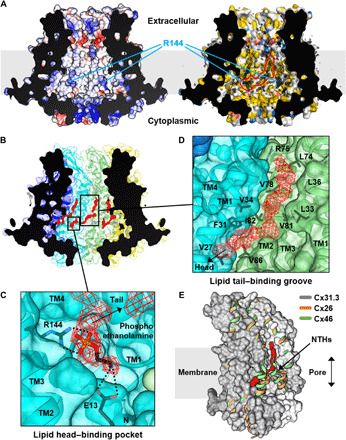
Putative lipid-binding sites on the inner surface of the channel pore. (**A**) Surface features of the pore inside are shown by vertical cross section of the hemichannel. The surface electrostatic potential (left) is calculated and colored using Adaptive Poisson-Boltzmann Solver (APBS). The displayed potentials range from −10 (red) to +10 (blue) *kT*/e. The surface hydrophobicity (right) is represented using PyMOL YRB script. The density maps inside the pore are shown in black meshes. The density blobs predicted to be a phospholipid molecule are encircled by red lines. The locations of the conserved R144 are indicated by cyan lines. (**B**) Prominently long density chains (red) are shown on the surface of the pore inside. (**C**) Close-up view of the shorter density chain. R144 and E13 are shown in sticks. A phosphoethanolamine molecule is modeled into the density. The black dotted lines indicate putative salt bridges. (**D**) Close-up view of the longer density chain. The density chain is located in a long hydrophobic groove formed in the interprotomer interface. The groove-lining residues, which are highly conserved in the connexin family (fig. S1), are shown in sticks and labeled. (**E**) NTHs in Cx26 and Cx46/50 GJIChs are located on the corresponding surface to the putative lipid-binding sites of Cx31.3 hemichannel. The Cx26 (orange ribbons) and Cx46 (green ribbons) GJICh structures were superposed on the Cx31.3 hemichannel structure (gray surface). Only two neighboring protomers for each channel are shown.

The large surface area inside the pore is mostly hydrophobic, indicating that this region should be covered by hydrophobic or amphipathic molecules in the purified protein sample (Fig. 2A). We identified long chains of map density on the hydrophobic surface, which likely correspond to lipid or detergent molecules (Fig. 2B). A strong density blob is located next to R144 in a deep hydrophilic pocket at the boundary of cytoplasmic and transmembrane regions, which spatially fit well at the head group of phosphatidylethanolamine (PE). In our putative PE-bound model, the phospho and amino groups of PE interact with the guanidino group of R144 and the carboxyl group of E13, respectively (Fig. 2C). We excluded the possibility that this density blob corresponds to LMNG because the binding pocket is too small for the detergent head group. It appears that phospholipids from human cell membrane have been strongly bound to the surface and not completely removed during purification. R144 is highly conserved in 20 human connexins, suggesting that its interaction with phospholipids may play a generally important structural role in these family proteins. This idea is supported by a previous study showing that mutations of R144 in Cx26 hemichannel considerably affect its permeability (*30*). The phospholipid tail seems to be located in a long hydrophobic groove formed by interprotomer interaction, suggesting that lipid binding may affect connexin assembly into hemichannel and vice versa (Fig. 2D). In the Cx26 and Cx46/50 GJICh structures, amphipathic NTHs interact with the corresponding region to the putative lipid-binding sites of Cx31.3 hemichannel, masking a large area of the hydrophobic pore surface and creating a more hydrophilic pore environment (Fig. 2E).

### Cx31.3 hemichannel with an ~8-Å-diameter pore is selectively permeable to chloride ions

**Fig. 3:**
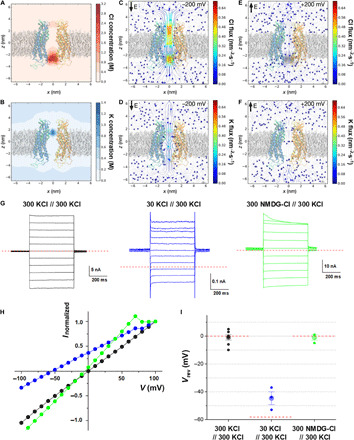
Anion-selective transport through Cx31.3 hemichannel. (**A** and **B**) Local concentration maps of Cl^−^ (A) and K^+^ (B) ions. Concentration maps were computed using the last 1-μs trajectory without a bias potential. (**C** and **D**) Ionic fluxes of Cl^−^ (C) and K^+^ (D) ions under a transmembrane potential of −200 mV. The total ionic currents at −200 mV is −0.166 nA. (**E** and **F**) Ionic fluxes of Cl^−^ (E) and K^+^ (F) ions under a transmembrane potential of +200 mV. The total ionic currents at +200 mV is +0.017 nA. (**G**) Representative current traces of wild-type Cx31.3 hemichannels with indicated ion gradients (in millimolar concentration, cis side//trans side) across the bilayer. Ionic currents were evoked by 500-ms test pulses from −100 to +100 mV, with 10-mV increments. For clarity, currents are displayed in 20-mV increments. Red dashed lines mark zero-current level. (**H**) Current-voltage relation (*I*-*V*) curves in a symmetrical KCl (black circle), 10-fold KCl gradient (blue circle), or asymmetrical cation gradient (green circle). Currents averaged between 290 and 490 ms into the test pulses are normalized to the values at +100 mV. (**I**) Reversal potentials (*V*_rev_) were determined by null-point measurement in a symmetrical KCl (black circle; *n* = 7), 10-fold KCl gradient (blue circle; *n* = 3), or asymmetrical cation conditions (green circle; *n* = 3). The Nernst potentials for the Cl^−^ (red dashed lines) are displayed in the indicated gradient. Each bar represents the means ± SEM.

In the Cx31.3 hemichannel structure, the cytoplasmic pore formed by N termini has a solvent-accessible diameter of ~8 Å (Fig. 1, B and F), which is slightly bigger than the effective hydrated diameters of K^+^ (~6.6 Å), Na^+^ (~7.2 Å), and Cl^−^ (~6.6 Å) (*31*). Therefore, we tested the ion permeability and selectivity of Cx31.3 hemichannel using MD simulations. In the 1.5-μs simulation at 150 mM KCl without transmembrane potential, we found that Cl^−^ ions gather at the cytoplasmic entrance, markedly increasing its local concentration up to ~3 M because of many basic residues in NTHs (Fig. 3A), while K^+^ ions localize to the inner pore surface with many acidic residues between extracellular and transmembrane regions, resulting in a local concentration of ~1 M (Fig. 3B). This notably asymmetric ionic distribution suggests that the ionic current through the channel pore may be selective and/or direction dependent. To address this hypothesis, we performed two additional simulations under −200- and +200-mV transmembrane potentials. At −200-mV bias, the Cl^−^ current (−0.131 nA) was about four times higher than the K^+^ current (−0.035 nA), indicating the selectivity for Cl^−^ over K^+^ (Fig. 3, C and D). The ionic currents at +200-mV bias for Cl^−^ (0.016 nA) and K^+^ (0.001 nA) also showed a strong selectivity for Cl^−^ (Fig. 3, E and F). The total ionic current at −200 mV (−0.166 nA) is 10 times bigger than that at +200 mV (0.017 nA), suggesting that Cx31.3 hemichannel is rectifying inwardly.

Next, we performed electrophysiological experiments to confirm the anion selectivity of Cx31.3 hemichannel shown in MD simulations. The hemichannel activity was examined by the planar lipid bilayer recording (*32*, *33*) with various ion compositions across the bilayer membrane: The Cx31.3 hemichannels were inserted into the bilayer membrane, and a series of voltage stimulations from −100 to +100 mV were applied to the bilayer (Fig. 3, G and H). Although the Cx31.3 hemichannels are inserted into the bilayer membrane in random orientation, the relative permeability can be obtained by the Goldman-Hodgkin-Katz (GHK) equation with the directly measured reversal potential, the zero-current voltage (see Materials and Methods). In the 10-fold KCl gradient (cis side, 30 mM KCl; trans side, 300 mM KCl; where the trans-side potential is 0 mV), the Nernst potentials for Cl^−^ and K^+^ are −58.2 and +58.2 mV, respectively. Since the reversal potential (*Vr*) is −44.7 ± 4.6 mV (*n* = 3; Fig. 3I) in the 10-fold KCl gradient, the relative permeability of potassium ion over chloride ion (*P*_K_/*P*_Cl_) is 0.08 ± 0.03. Under the asymmetrical cation conditions [cis side, 300 mM *N*-methyl-d-glucamine (NMDG)–Cl; trans side, 300 mM KCl], the *Vr* (−1.0 ± 2.0 mV; *n* = 3) is closed to the Nernst potential of Cl^−^ (0 mV at symmetrical 300 mM Cl^−^), and the permeability of NMDG over potassium ion (*P*_NMDG_*/P*_K_) is 1.04 ± 0.09 (Fig. 3I). These results indicate that the Cx31.3 hemichannel favors to transport Cl^−^ ~12.5 times more than cation and shows a marginal selectivity between cations. However, it should be noted that the relative permeability of ions calculated from the GHK equation needs to be interpreted with caution because the equation stands on the two essential assumptions: the independent transport of ions through the pore and the linear voltage drop across the membrane. In the MD simulations of Cx31.3 hemichannel, K^+^ and Cl^−^ appear to be locally concentrated at the extracellular and cytoplasmic entrances of the pore, respectively (Fig. 3, A and B), and this would induce nonlinear voltage drop across the membrane. Therefore, the above calculated relative permeabilities of Cx31.3 hemichannel may have errors caused by inconsistent electric field in the membrane. Nevertheless, from the reversal potential measurements, we could still conclude that anions move through the pore more favorably than cations. To further examine the selectivity between anions, the relative permeability between Cl^−^ and a polyatomic anion, isethionate (HO-CH_2_-CH_2_-SO_3_^−^), was accessed by asymmetric anion gradient (cis side, 10 mM KCl and 290 mM K-isethionate; trans side, 290 mM KCl and 10 mM K-isethionate), where the Nernst potentials of Cl^−^ and isethionate are −85.1 and 85.1 mV, respectively. The reversal potential (−51.5 ± 3.5 mV; *n* = 4) (fig. S4, A to C) indicates that the Cx31.3 hemichannel has 10 times higher permeability of Cl^−^ than a polyatomic anion, isethionate (*P*_isethionate_/*P*_Cl_ = 0.10 ± 0.02).

Together, these findings suggest that Cx31.3 hemichannel in the entrance-covering NTH conformation is selectively permeable to small anions and likely exports anions rather than imports cations. They also imply that no conformational change of NTHs would be necessary for this activity, although the conformational change may be able to further increase the activity.

### The structure of the R15G-mutant hemichannel

**Fig. 4:**
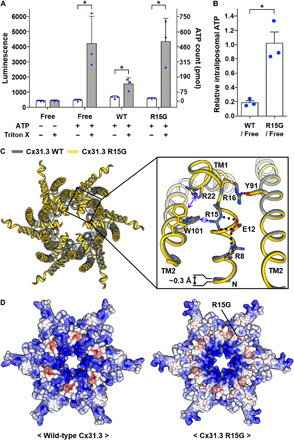
Comparison between wild-type and R15G-mutant hemichannels. (**A**) ATP transport assay for wild-type (WT) and R15G-mutant Cx31.3 hemichannels. ATP-loaded liposomes with or without Cx31.3 hemichannels were subjected to desalting column chromatography. Extraliposomal and total ATPs in 10 μl of the eluted liposome fraction were measured by the luciferase assay and represented as white and gray bars, respectively. ATP loading before and Triton X-100 treatment after the desalting process were indicated below. The data are expressed as the means ± SD of the luminescence values from three independent experiments. The asterisks denote *P* < 0.05 compared between the indicated measurements. (**B**) Relative amounts of ATPs retained inside proteoliposomes compared with that inside protein-free liposomes. The intraliposomal ATP count was calculated from extraliposomal and total ATP counts in (A). The data are expressed as the means ± SE. (*n* = 3). The asterisk denotes *P* < 0.05 compared between the indicated values. (**C**) WT (gray) and R15G-mutant (yellow) hemichannels were superimposed and shown from cytoplasmic view. The box indicates close-up view of the region around NTHs. The residues showing substantial conformational changes by R15G mutation are represented in sticks. The NTH of the mutant Cx31.3 is a little stretched toward the pore center, compared with that of WT Cx31.3. The black dotted lines indicate salt bridges and hydrogen bonds. The blue lines indicate cation-π interactions. (**D**) The electrostatic potential of the cytoplasmic surface of WT hemichannel (left) is compared with that of R15G mutant (right). The APBS was used to calculate the electrostatic potential at pH 7. The displayed potentials range from −10 (red) to +10 (blue) *kT*/e. The location of R15G is indicated.

A previous study has shown that HeLa cells stably expressing Cx31.3 release ATPs about three times more than normal HeLa cells or those stably expressing the R15G variant of Cx31.3 (*29*). To confirm the ATP transport activity of Cx31.3 hemichannel and the inhibitory effect of R15G mutation, we performed the ATP release assay using purified hemichannels. We reconstituted wild-type and R15G-mutant hemichannels into liposomes and loaded ATPs into proteoliposomes by the freeze-thaw method. We removed ATPs outside of the liposomes by desalting column chromatography and measured the ATP level inside the liposomes. The result showed that wild-type proteoliposomes, mutant proteoliposomes, and protein-free liposomes retained ~100, ~500, and ~500 pmol of ATPs, respectively (Fig. 4, A and B). This indicates that wild-type proteoliposomes released most ATPs during the desalting process, while mutant proteoliposomes retained ATPs in the similar level as free liposomes.

Since both the previous cell-based study (*29*) and our in vitro assay showed that R15G mutation strongly inhibits the ATP release by Cx31.3 hemichannel, we thought that the R15G-mutant hemichannel would adopt the closed conformation for at least molecules of which sizes are similar to or bigger than that of ATP. Therefore, we solved the cryo-EM structure of the R15G-mutant hemichannel in the same buffer and detergent environments, as used for the wild-type hemichannel (fig. S5). Compared with the wild-type hemichannel structure, the R15G mutant showed some changes in the polar interaction network between NTH and TM2 (Fig. 4C) and notably decreased the basicity of the cytoplasmic surface (Fig. 4D). However, we found no major structural change and almost no change in the pore diameter at the cytoplasmic entrance by this mutation (Fig. 4C). This, together with the ATP transport assay, suggests that ATP molecules cannot freely diffuse through the mutant hemichannel in the entrance-covering NTH conformation. Because the pore diameter (~8 Å) at the cytoplasmic entrance is much smaller than the effective hydrated diameter of ATP (~12 Å) (*34*), this conformation would not allow ATP to move through the pore without substantial conformational changes of NTHs. However, it is still unclear how the wild-type hemichannel, but not the mutant, could change the NTH conformation to transport ATP.

### The hemichannel structure in the presence of calcium ions reveals the Ca^2+^-binding tunnel

The outer end of the transmembrane pore in Cx31.3 hemichannel forms an acidic band (Fig. 2A), which is highly conserved in 20 human connexins. The corresponding region in Cx26 hemichannel or GJICh is known to be crucial for gating and permeability regulation by Ca^2+^ ions (*13*, *26*). Water molecules with clear map densities are concentrated on this surface and in ECLs, which show a relatively higher structural flexibility than transmembrane domains (TMDs) (fig. S6). These data collectively suggest that extracellular Ca^2+^ ions may replace several water molecules around the acidic band to change the surface electrostatics and play a structural role.

**Fig. 5:**
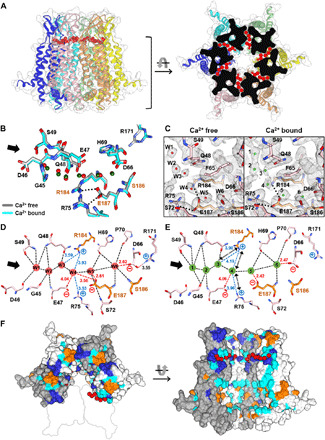
The Ca^2+^-binding tunnel. (**A**) Water molecules in the Ca^2+^-binding tunnel are shown as red spheres in the ribbon drawings of Cx31.3 hemichannel structure in the absence of Ca^2+^. (**B**) The tunnel-lining residues in the structurally aligned Ca^2+^-free (gray) and Ca^2+^-bound (cyan) hemichannels are represented in sticks and are labeled. F65, P70, L71, S72, P185, and K188 are not shown for clarity. Six water molecules in the tunnel of the Ca^2+^-free structure are shown in red spheres. Putative calcium ions in the tunnel of the Ca^2+^-bound structure are shown in green spheres. The black arrow indicates the tunnel entrance. (**C**) Comparison between the density maps of the Ca^2+^-binding tunnel in the presence and absence of calcium ions. Both maps are contoured at σ = 5.3. Two neighboring connexins in the hemichannel are colored pink and orange, respectively. The side chains of the residues are represented in sticks. (**D** and **E**) Schematic diagrams of the Ca^2+^-binding tunnel in the absence (D) and presence (E) of calcium ions. All tunnel-lining residues are shown and colored, as in (C). Dashed lines indicate the interactions of the tunnel-lining residues with water molecules (D) or putative calcium ions (E) in the distances equal or less than 4 Å. The interaction distances involving charged residues are labeled. Arrows indicate potential repulsion between calcium ions and basic residues. (**F**) On the surface representations of Cx31.3 hemichannel, 100% identically conserved residues among 20 human connexins are colored blue, >90% identically or 100% similarly conserved residues are colored cyan, and Cx31.3-specific residues are colored orange. Four of six connexin protomers are shown for clarity.

To identify Ca^2+^-binding sites and understand Ca^2+^-dependent regulatory mechanism, we determined Cx31.3 structure in the presence of 50 mM Ca^2+^ at 2.5-Å resolution (fig. S7) and compared it with that in Ca^2+^-free state. While the overall structure did not change with Ca^2+^ ions, we found small but substantial changes in map densities in a deep tunnel at the interprotomer interface around the acidic band (Fig. 5A). In the Ca^2+^-free state, the tunnel is occupied by six water molecules (W1 to W6) with clear map densities, which form an extensive interaction network with the tunnel-lining residues (Fig. 5, B to D). In the structure with Ca^2+^ ions, although we could not distinguish Ca^2+^ densities from water densities due to large overlaps of Ca^2+^ and water positions, we could observe substantial changes in map densities in and around the Ca^2+^-binding tunnel. First, the density blobs corresponding to W1 to W4 are considerably smeared, indicating that the water-mediated interaction network is disturbed by Ca^2+^ ions (Fig. 5, B, C, and E). In particular, the W4 position is slightly relocated further from R75 and R184, and the side-chain conformation of E47 is largely changed and more stabilized, as judged by a bigger and clearer density of E47 in the presence of Ca^2+^ ions (Fig. 5, D and E, and fig. S8A). This is likely caused by the interaction between Ca^2+^ and E47 and the repulsion from the two arginine residues. Second, a relatively smaller change, however, was observed for W5, although W5 closely interacts with E187 (fig. S8B). Ca^2+^ seems to be weakly bound to this residue because its negative charge is partly neutralized by the interaction with R75 and R184. Third, the map densities for W6, D66, and R171 show detectable changes between two structures in the presence and absence of Ca^2+^ ions (fig. S8, B and C), suggesting that the charge neutralization of D66 through Ca^2+^ binding weakens the interaction between D66 and R171.

Highly conserved residues in 20 human connexins are concentrated in the Ca^2+^-binding tunnel. Eight of 17 tunnel-lining residues are identically conserved residues, and 6 residues are 90% identically or 100% similarly conserved (Fig. 5F and fig. S1). Moreover, a number of disease-causing mutations in various connexins are found in the conserved Ca^2+^-binding tunnel (*35*). These data strongly suggest that intact Ca^2+^-binding tunnels are functionally crucial for most connexin family members.

### Conformational changes in and around the Ca^2+^-binding tunnel between hemichannel and GJICh structures

**Fig. 6:**
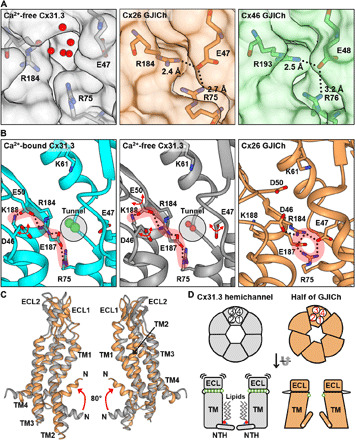
Detailed structural comparison of Cx31.3 hemichannel with GJIChs. (**A**) The Ca^2+^-binding tunnel is open in Cx31.3 hemichannel (left) but closed in Cx26 [Protein Data Bank (PDB ID), 2ZW3] (middle) and Cx46 (PDB ID, 6MHQ) (right) GJIChs. Cx31.3 hemichannel, Cx26 GJICh, and Cx46 GJICh are colored in gray, orange, and green, respectively. The black dotted lines indicate salt bridges. Red spheres represent water molecules in the tunnel. (**B**) Salt-bridge networks in and around Ca^2+^-binding tunnels of Ca^2+^-bound Cx31.3 hemichannel (left), Ca^2+^-free Cx31.3 hemichannel (middle), and Cx26 GJICh (right). The salt bridge–forming residues are shown in sticks and labeled. Salt bridges with distances less than 4 Å are indicated by black dotted lines. Transparent gray circles indicate Ca^2+^-binding tunnels. Red and green spheres represent water molecules and putative Ca^2+^-binding positions, respectively. Red two-way arrows represent high flexibility of the indicated residues. The linear and circular salt-bridge networks are highlighted by transparent red marking lines. (**C**) Structural differences in ECLs and TMDs between Cx31.3 hemichannel (gray) and Cx26 GJICh (orange). The overall structures of Cx31.3 and Cx26 channels were aligned, and two interfacing connexins in each channel are shown. Red arrows indicate substantial conformational shifts of NTHs in Cx26 GJICh compared to Cx31.3 hemichannel. (**D**) Schematic diagrams represent overall conformational differences between Cx31.3 hemichannel and Cx26 GJICh. Extracellular (top) and side (bottom) views are shown. Circled numbers indicate TM helices. Green circles indicate Ca^2+^-binding sites shown in the current Cx31.3 structure and the Ca^2+^-bound Cx26 GJICh structure (PDB ID, 5ER7).

Since the Ca^2+^-binding tunnel is the most highly conserved region in the connexin family, we compared the structure in and around the tunnel of Cx31.3 hemichannel with those of previous GJICh structures. In both Cx26 and Cx46/50 GJICh structures, the Ca^2+^-binding tunnel is completely blocked by the corresponding residues to E47, R75, and R184 of Cx31.3 (Fig. 6A). These residues, together with the corresponding residues to E187 and D46 of Cx31.3, form a circular salt-bridge network (Fig. 6B, right, and fig. S9A), which contrasts with the linear network in the Cx31.3 hemichannel involving R75, E187, R184, E50, and K188 in the order (Fig. 6B, left and middle). The residues involved in circular and linear salt-bridge networks, except E50, are nearly completely conserved among 20 human connexins (fig. S1). In the detailed comparison between Cx26 and Cx31.3, the interactions of E187 with R75 and R184 are maintained in both circular and linear networks. For Cx26, however, D46 and E47 join the central R75-E187-R184 interaction, and D50 and K188 are distantly located from the central interaction and vice versa for Cx31.3 (Fig. 6B, middle and right). In addition, D50 in Cx26 GJICh appears to intermolecularly interact with K61, and this interaction is important for stabilizing the open state of Cx26 hemichannel (*13*), whereas E50 in Cx31.3 hemichannel is distantly located from K61 (Fig. 6B).

The closed tunnel and the circular salt-bridge network in GJICh are also linked to the conformational differences of ECLs and TM helices. When the Cx31.3 hemichannel structure is superposed with the hexameric halves of the Cx26 or Cx46/50 GJICh structures, both GJIChs show slight rotation and tilting of TMDs and ECLs compared to the hemichannel (Fig. 6C and fig. S9B); however, the superposition of individual protomers shows no substantial conformational difference in TM helices between hemichannel and GJIChs (fig. S9C).

## DISCUSSION

The hemichannel structure showed that the entrance-covering NTH conformation forms a small pore slightly bigger than hydrated K^+^ or Cl^−^ ions, suggesting that the transport of metabolites much bigger than ions would require a conformational change of NTH. This idea is further supported by the ATP-impermeable R15G-mutant hemichannel structure that also adopts a similar NTH conformation. In addition, the electrophysiological experiments showed that isethionate, of which size is similar with that of pyruvate, cannot efficiently pass through the hemichannel. Therefore, we propose that the entrance-covering NTH conformation represents a partially closed state of Cx31.3 hemichannel, which would prevent the movement of metabolites through and selectively allows small anions to pass through the hemichannel. Since one of the main functions of GJIChs is the metabolic synchronization between adjacent cells, GJIChs are expected to be open under normal conditions and closed in response to specific signals such as increased Ca^2+^ level. Hemichannels, in contrast, are closed when not stimulated to prevent unwanted release of intracellular metabolites (*7*, *36*). Although the Cx31.3 hemichannel structure does not exhibit the completely closed state, it explains how hemichannels could block the nonspecific transport of cellular metabolites. However, it is still unclear whether other connexin hemichannels adopt similar NTH conformations as Cx31.3 since the NTH-TM2 interaction in Cx31.3 hemichannel is moderately conserved in only half of 20 human connexin family members. Although the substrate selectivity can change, depending on sequences and structures of not only NTH but also CL and C-terminal loop (CTL), the moderate or low conservation of the NTH-TM2 interaction would be at least partly related to highly diverse permeation/transport characteristics reported for different connexin hemichannels (*37*, *38*). For example, Cx43 hemichannel transports ATP but not glutamate and ions at the physiological membrane potential, while Cx30 hemichannel efficiently transports ATP, glutamate, and ions. Cx32 hemichannel cannot transport ATP but other signal molecules such as adenosine 3′,5′-monophosphate, guanosine 3′,5′-monophosphate, and inositol 1,4,5-trisphosphate. The question of whether other hemichannels adopt different NTH conformations or have similar NTH conformations but different gating mechanisms involving CL or CTL could be addressed by the structures of other connexin hemichannels.

The partially closed structure of Cx31.3 hemichannel contrasts the previous plug-gating model based on the Cx26 GJICh structure (*17*). There are two possible reasons for the difference between two models. First, the hydrophobic residues crucial for the NTH-TM2 interaction are conserved quite well in A-class connexins, including Cx43 and Cx46, but not in B-class connexins, including Cx26. Therefore, NTHs in Cx26 GJICh, which is released from the inner pore surface of TMD by M34A mutation, would not interact with TM2 and gather to form a plug in the pore inside. Second, both Cx26 and Cx46/50 GJICh structures show a gradual increase in flexibility from the extracellular to the cytoplasmic end of TMD, while the Cx31.3 hemichannel structure does not (fig. S6B). The increased structural flexibility of the cytosolic region in Cx26 GJICh may reduce the binding affinity between NTH and TM2. In this case, the NTH-gating mechanism would differ not between A and B classes but between GJICh and hemichannel states.

We have found putative lipid-binding sites on the inner pore surface of Cx31.3 hemichannel. TM1 and TM2 of all connexin family proteins are similarly hydrophobic (fig. S1). Therefore, the dissociation of NTHs from TM1 and TM2 helices in their hemichannel or GJICh states necessarily exposes a large area of hydrophobic surface in the inner pore. If their gating mechanisms involve the movement of NTHs, then such a relatively unstable state can be more stabilized by lipid binding to the hydrophobic surface to maintain a different NTH conformation such as the entrance-covering conformation of Cx31.3 hemichannel (Fig. 2). Consistent with this notion, a recently reported structure of innexin-6 hemichannel in a lipid nanodisc showed that the hemichannel pore is filled with lipids, and six NTHs are collocated at the entrance similarly, as shown in the Cx31.3 hemichannel structure (*39*). This report suggests that lipid molecules in the hemichannel pore cavity play an important role in the hemichannel closing. It is, however, unclear how lipids leave the pore cavity for the opening or during the connexon-connexon docking. In innexin-6 hemichannel, lipid molecules could go through a large cleft between two interfacing TMDs. However, such a hole is not seen in the Cx31.3 hemichannel structure and available GJICh structures, and, therefore, it remains to be addressed whether and how lipids enter and exit the pore during the hemichannel closing and opening.

In the hemichannel structure with Ca^2+^ ions, we have not observed close coordination by more than one residue but found many putative binding sites, especially in the Ca^2+^-binding tunnel. This suggests that Ca^2+^ ions may play its inhibitory role via multiple weak interactions with functionally important acidic residues, including E47, D66, and E187. Although Ca^2+^ ions may not strongly bind to E47 and E187 in the tunnel, the dynamic interaction between these residues and Ca^2+^ ions in the tunnel could disturb the interprotomer interactions, including water-mediated ionic interaction between E47 and R184 and the salt bridge between E187 and R75.

In addition, the interaction of D66 with R171 appeared to be weakened by Ca^2^ binding. Since R171 is located close to the head group of LMNG in Cx31.3 structure (fig. S10), the head group of phospholipid in the physiological membrane may directly interact with this residue, and the interaction may be stronger in the presence of Ca^2+^ ions. D66H mutation in Cx26, which is known to inhibit intercellular communication (*40*, *41*), may mimic Ca^2+^-bound state to prevent GJICh formation.

Our comparative structural and sequence analysis of Cx31.3 hemichannel, Cx26 GJICh, and Cx46/50 GJICh showed notable structural differences in not only NTHs but also ECLs and TMDs. Although we cannot exclude the possibility that the structural features of hemichannel are only specific for Cx31.3, high conservation of most residues important for the structural changes suggests that other connexin hemichannels may have a similar conformation with the Cx31.3 hemichannel. If this is true, then during their connexon-connexon docking, two interfacing hemichannels would undergo a series of conformational changes in ECLs, Ca^2+^-binding tunnels, and TMDs (Fig. 6D).

Several studies on gap junctions in fiber cells of calf lens and heart muscle cells have demonstrated that gap junction formation is inhibited by Ca^2+^ ions (*42*, *43*). In the Cx31.3 structure with Ca^2+^ ions, the side-chain conformations of E47 and E50 appear to be more stabilized than that in Ca^2+^-free structure, as judged by bigger and clearer densities of these residues (Fig. 6B, left and middle, and fig. S8, A and D), suggesting that Ca^2+^ ions may inhibit the circular salt-bridge network and stabilize the linear network, consequently preventing gap junction formation of other connexin family members. However, although the Ca^2+^-binding tunnel is highly conserved through the connexin family, this hypothesis is based on the assumption that other connexins have similar hemichannel structures as Cx31.3 and, therefore, needs to be confirmed by the structures of other hemichannels.

The crystal structure of Cx26 GJICh in the presence of Ca^2+^ ions [Protein Data Bank (PDB) ID, 5ER7] has also shown that a Ca^2+^ ion is bound to E47 on the surface of the GJICh pore (*26*). While this binding appears to disturb the circular salt-bridge network, it could not cause any conformational change in the Ca^2+^-binding tunnel and ECLs. The Cx31.3 hemichannel structure in the presence of Ca^2+^ ions also showed no conformational change, except for several side-chain conformations, compared with its Ca^2+^-free structure. This suggests that the permeant selectivity could be changed by extracellular Ca^2+^ ions through the positive electrostatic barrier mechanism, which is proposed on the basis of the Ca^2+^-bound Cx26 GJICh structure (*26*). Ca^2+^ binding seems to be not strong enough to drive a large conformational change of hemichannel or GJICh at least in detergent environments, and an additional factor, possibly lipids, may be required for the complete channel closing by Ca^2+^ binding.

E50, which participates in the linear salt-bridge network around the Ca^2+^-binding tunnel of Cx31.3 hemichannel, is conserved as acidic residues in only 12 human connexin members, while the other four residues in the linear network are almost identically conserved in all 20 human members. Therefore, the full linear network is not likely necessary for the functions of at least eight connexin members. For these eight members, the dissociation of the corresponding residues to E47 from the circular salt-bridge network may be sufficient for the opening of the Ca^2+^-binding tunnel and the subsequent structural change in ECLs.

In the sequence alignment of 20 human connexins, we found that Cx31.3 has 12 unique residues to which the corresponding residues in other family members are conserved as different residues (Fig. 5F and fig. S1). Since these residues are located far from Ca^2+^ tunnel (eight residues on the inner or outer pore surface of the cytoplasmic half of TMD, three on the extracellular surface, and one at the N-terminal region), they would not directly affect the structural dynamics in and around Ca^2+^ tunnel. Notably, N54 and D179 in Cx26, which are critical for the connexon-connexon docking, are replaced with H54 and T179 in Cx31.3, suggesting that these substitutions are the major reason why Cx31.3 cannot form GJICh.

The Cx31.3 hemichannel structure showed the overall conformation that is notably different from the previous structures of hexameric halves of GJIChs. Although there is a high possibility that all connexin family proteins also adopt a similar conformation in their hemichannel states due to high sequence similarity among them, we should be aware that the structure may be specific for Cx31.3 because Cx31.3 hemichannels do not form GJIChs, are expressed specifically in oligodendrocytes, and may have a specialized function of ion transport. In addition, since the presented three structures similarly exhibit a partially closed conformation, we still lack understandings on the open state for ATP release and the completely closed state of hemichannel. Compared with the Cx31.3 hemichannel conformation, the completely closed state may have a different ECL conformation, as proposed in the previous loop-gating model (*44*, *45*) or a similar channel conformation with phospholipids occluding the pore cavity (*39*). More structural studies on various connexin hemichannels in lipid environments would be necessary to address these questions.

## MATERIALS AND METHODS

### Cloning, expression, and purification of Cx31.3 hemichannels

The coding sequence of human Cx31.3 or Cx31.3-R15G fused to the C-terminal 10His-tagged enhanced yellow fluorescent protein (eYFP) was respectively inserted into the pX vector (*46*). The expression plasmid was amplified in *Escherichia coli*, purified, and transfected into human embryonic kidney–293E cells that were grown in suspension in Ca^2+^-free Dulbecco’s modified Eagle’s medium (DMEM) supplemented with 5% fetal bovine serum at 37°C. Dimethyl sulfoxide (Amresco) was added immediately after transfection to the final concentration of 1%, and tryptone (Amresco) was added 24 hours after transfection to the final concentration of 0.5%. The transfected cells were harvested 48 hours after transfection.

All purification steps were carried out at 4°C or on ice unless indicated otherwise. The transfected cells in 1.5 liter of culture were harvested and resuspended in 50 ml of buffer A [20 mM caps (pH 10.5), 250 mM NaCl, and 2 mM β-mercaptoethanol] supplemented with 10% glycerol, 1 mM EDTA, 1 mM phenylmethylsulfonyl fluoride (PMSF), and one tablet of EDTA-free Pierce protease inhibitor tablets (Thermo Fisher Scientific, catalog no. 88666). The resuspended cells were lysed using a Dounce homogenizer (Bellco) with a loose (A) pestle, and the membrane fraction was isolated by high-speed centrifugation at 50,000*g* for 1 hour. The resulting membrane pellets were resuspended using a WiseTis homogenizer (DAIHAN Scientific) in 50 ml of buffer A supplemented with 1 mM PMSF, one tablet of EDTA-free Pierce protease inhibitor tablets (Thermo Fisher Scientific, catalog no. 88666), and 0.4% (w/v) LMNG (Anatrace). After incubation with a slow rotation for 90 min, the sample was mixed with 2.5 ml of neutralization buffer containing 1 M Hepes (pH 7.0), 500 mM NaCl, and 2 mM β-mercaptoethanol to lower the sample pH up to ~7.5 and centrifuged at 50,000*g* for 1 hour. The supernatant was loaded onto a Ni–nitrilotriacetic acid (NTA) column that was equilibrated with buffer B [20 mM Hepes (pH 7.5), 250 mM NaCl, 2 mM β-mercaptoethanol, and 0.004% LMNG]. After the column was washed with buffer B supplemented with 40 mM imidazole (Sigma-Aldrich), the bound proteins were eluted with buffer B supplemented with 200 mM imidazole. To remove imidazole, the eluted protein samples were loaded on a HiPrep 26/10 desalting column (GE Healthcare) equilibrated with buffer B. The proteins in buffer B were treated with a human rhinovirus 3C protease overnight to remove eYFP-10His. The protease-treated samples were loaded onto a Ni-NTA column again to separate hCx31.3 from the His-tagged proteases, eYFP-10His, and other protein impurities with substantial Ni-binding affinity. About half of hCx31.3 proteins were flown through the column, but the other half was weakly bound to the resin and, thus, eluted with buffer B supplemented with 20 mM imidazole. The unbound and eluted fractions were concentrated to ~4 mg/ml using an Amicon ultracentrifugal filter (molecular weight cutoff, 100 kDa), filtered with a 0.22-μm filter, and further purified using a Superdex 200 Increase 10/300 GL column (GE Healthcare) equilibrated with buffer B. The peak fraction (0.8 mg/ml for wild-type Cx31.3 and 0.6 mg/ml for its R15G mutant) was flash frozen in liquid nitrogen and stored at −80°C. Protein purity and quality were assessed by SDS–polyacrylamide gel electrophoresis and negative-stain EM.

### Identification of N-terminal modification

Purified Cx31.3 proteins were treated with Glu-C endoproteinase and analyzed by mass spectrometry in the Proteomics Core Facility in the Center for RNA Research (Institute for Basic Science and Seoul National University). From the mass spectrometry data, we searched for the molecular masses of N-terminal peptides with methionine deletion, acetylation, propionylation, and/or palmitoylation at the N terminus. We found that Met^1^ was completely removed, and Cys^2^ was partially acetylated. No propionylation or palmitoylation at Cys^2^ was observed.

### Sample vitrification, data collection, and image processing for 3D reconstruction

Four microliters of Cx31.3 hemichannel samples (1 mg/ml for wild-type Cx31.3 and 0.6 mg/ml for R15G mutant) was applied onto positively glow-discharged holey carbon grids (Quantifoil R1/2 Cu 300 mesh, SPI). The grids were blotted for 7 s using Vitrobot Mark IV (Thermo Fisher Scientific, USA) at 4°C with 100% humidity and plunge frozen in liquid ethane cooled by liquid nitrogen. For preparation of the hemichannel sample in the presence of Ca^2+^ ions, 1 M CaCl_2_ stock solution was added into the purified protein sample (1 mg/ml) to a final concentration of 50 mM, and the sample was incubated for 1 hour before vitrification.

Cryo-EM images of frozen-hydrated Cx31.3 hemichannel particles were collected at Korea Basic Science Institute using a Titan Krios (Thermo Fisher Scientific, USA) transmission electron microscope operated at 300 kV. Movie data were recorded using a Falcon 3EC direct electron detector (Thermo Fisher Scientific, USA) using electron counting mode and automatic data acquisition software (EPU; Thermo Fisher Scientific, USA). Detailed data acquisition conditions and parameters are provided in table S1.

Cryo-EM images of wild-type Cx31.3 hemichannel were processed using RELION 3.0 (*47*) unless otherwise stated. Beam-induced motion correction and dose weighting were performed using MotionCor2 version 1.2.1 (*48*), and contrast transfer function (CTF) estimation was performed using Gctf version 1.06 (*49*). Micrographs unsuitable for image processing such as those containing extremely low or high defocus and large motion drifts were removed by manual inspection. Then, 1,479,056 particle images were semiautomatically picked from 3284 micrographs and extracted into 320 pixel boxes. Six rounds of two-dimensional (2D) classifications were carried out to eliminate poorly aligned particles, and 846,910 particles were lastly selected for further processing. Then, three successive 3D classifications were performed, and the 3D initial model from 2D particle images was generated by a stochastic gradient descent algorithm in RELION. Next, images with defocus values lower than the arbitrarily chosen value of −1.6 μm were selected for subsequent steps because such low-defocus images are likely to contribute to high-resolution information in the final reconstruction (*50*). Assorted 286,968 particles were included in the final 3D refinement with C1 and C6 symmetry impositions. Following the refinement, per-particle CTF estimation and motion correction were performed using RELION’s CTF refinement and particle polishing regime (*51*). Last, the map was sharpened upon applying tight mask and using unfiltered half maps. The estimated resolution of the final map was 2.34 Å based on the 0.143 Fourier shell correlation (FSC) criterion (fig. S2B) (*52*). The dataset for hemichannel in the presence of Ca^2+^ was processed using similar routine. Five rounds of 2D classifications and four successive rounds of 3D classifications were performed to sort out “good” particle images. Assorted 185,517 particles were included in the final 3D refinement with C6 symmetry imposition. Following the refinement, per-particle CTF estimation and motion correction were performed using RELION’s CTF refinement and particle polishing regime (*51*). Last, the map was sharpened upon applying tight mask and using unfiltered half maps. The estimated resolution of the final map was 2.53 Å based on the 0.143 FSC criterion (fig. S7B) (*52*).

The dataset for Cx31.3-R15G–mutant hemichannel was processed using cryoSPARC version 2 (*53*). The recorded movies were subjected to motion correction and CTF estimation using full-frame motion correction and Gctf version 1.06 in cryoSPARC. Total 1,834,380 particle images were automatically picked and extracted into 320 pixel boxes. To select good particles with various orientations and high homogeneity, we performed four rounds of 2D classifications. After homogenous refinement for 3D reconstruction, local motion correction and further particle selection were carried out. During heterogeneous refinement, the dataset was divided into two 3D classes. The good class containing 205,646 particle images was used for the final homogenous refinement with C6 symmetry imposition. The map was sharpened by applying an inverse B factor of −100 Å (fig. S5B).

Detailed workflows and parameters are shown in figs. S2, S5, and S7, and table S1. The local resolutions of 3D maps were estimated using ResMap (*54*).

### Model building and refinement

The structural models for Cx31.3 hemichannel were built manually in the Coot program and refined using phenix.real_space_refine in the PHENIX software suite (*55*). The final models contain LMNG detergents and do not contain Met^1^ and N-terminal acetylation (see the main text for details). The final models for wild-type Cx31.3 with or without Ca^2+^ ions and R15G mutant do not include residues 108 to 126 in the CLs and residues 222 to 279 in the CTLs of which map densities are missing. The geometric restraints for LMNG detergents were optimized using the eLBOW module in PHENIX. For validation, FSC curves were calculated between the final models and EM maps in figs. S2, S5, and S7. The quality of final models was evaluated using the “comprehensive model validation” section and MolProbity in PHENIX. Detailed information for refinement and validation statistics was included in table S1. The pore radii were calculated using HOLE (*56*). The electrostatic potential of the surface was calculated using the Adaptive Poisson-Boltzmann Solver (APBS) (*57*) and PyMOL YRB script (*58*). Structures were visualized and figures were produced using the UCSF (University of California, San Francisco) Chimera and PyMOL programs.

### MD simulation protocol

All MD simulations were performed using the GROMACS 2018.2 package (*59*) and the CHARMM36m force field (*60*), combined with the CUFIX corrections for charge-charge interaction pairs (*61*, *62*). For water molecules, the CHARMM-modified TIP3P model was used (*60*). Temperature was kept constant at 300 K using the Nosé-Hoover scheme (*63*, *64*). To achieve a constant surface tension–constant temperature (NPγT) ensemble at zero surface tension (γ = 0), we semi-isotropically coupled the lateral (*x*-*y*) and normal (*z*) pressures to 1 bar using the Parrinello-Rahman scheme (*65*). For the evaluation of van der Waals forces, a 10- to 12-Å switching scheme was used. For the evaluation of long-range electrostatic forces under periodic boundary conditions, the particle-mesh Ewald summation scheme (*66*) was used; the grid spacing and the real-space cutoff were 1.2 and 12 Å, respectively. LINCS (*67*) and SETTLE (*68*) algorithms were used to constrain covalent bonds to hydrogen in nonwater and water molecules, respectively. In all simulations, the integration time step was 2 fs, and coordinates were recorded every 20 ps. The visualization scheme for the density-flux map in Fig. 3 (A to F) is described in a previous report by Yoo and Aksimentiev (*69*).

### MD preparation of the Cx31.3 hemichannel embedded in a lipid bilayer

Because the experimental hexamer structure of the Cx31.3 hemichannel lacks unstructured CLs (residues 108 to 126), we manually reconstructed the loop of each chain such that the reconstructed loops do not induce any clash. We placed the channel in a lipid bilayer of a 4:1 random mixture of 1-palmitoyl-2-oleoyl-*sn*-glycero-3-phosphatidylcholine (POPC) and 1-palmitoyl-2-oleoyl-*sn*-glycero-3-PE (POPE) and removed overlapping lipid molecules. We added six POPE lipid molecules in the internal cavity and paired each of which with a chain using two distance constraints, one between the C𝜁 atom of Arg^144^ and the lipid phosphorus atom and the other between the Cδ atom of Glu^13^ and the lipid amine nitrogen atom. For these distance constraints, we used the half-harmonic function that is effective at a distance higher than 8 Å. Last, we added an explicit solution of 150 mM KCl. The final system contained a Cx31.3 hemichannel, 192 POPC and 48 POPE lipids in the upper leaflet, 169 POPC and 40 POPE lipids in the lower leaflet, 55,712 water molecules, 190 K^+^ ions, and 220 Cl^−^ ions in a rectangular volume of about 140 × 140 × 130 Å^3^. After 5000-step energy minimization, we performed 80, 30, and 390 ns of equilibration using harmonic restraints on all nonhydrogen atoms of protein except unstructured CLs, with force constants of 1000, 100, and 0 kJ mol^−1^ nm^−2^, respectively. The system with a connexin protomer was prepared and equilibrated in a similar way. The monomer system contained a Cx31.3 monomer, 120 POPC and 30 POPE lipids in each leaflet, 3000 water molecules, 100 K^+^ ions, and 105 Cl^−^ ions in a rectangular volume of about 100 × 100 × 130 Å^3^.

### Preparation of proteoliposome and electrical recording of Cx31.3 hemichannels in planar lipid bilayers

Purified wild-type Cx31.3 proteins were mixed with phospholipids [POPE (15 mg/ml) (Avanti Polar Lipids) and 1-palmitoyl-2-oleoyl-phosphatidylglycerol (POPG; 5 mg/ml; Avanti Polar Lipids)] solubilized in 35 mM 3-[(3-cholamidopropyl)-dimethylammonio]-1-propane sulfonate (Anatrace) at various protein-to-lipid ratios (0.2 to 1 μg/mg). Proteoliposomes were formed by dialysis of the protein-lipid mix against the reconstitution buffer [20 mM Hepes (pH 7.5), 250 mM NaCl, and 2 mM β-mercaptoethanol] at room temperature for 72 hours with three buffer changes. After dialysis, the proteoliposomes were aliquoted and stored at −80°C. The proteoliposomes were freeze thawed for 3 cycles before used in the bilayer recording. Ionic currents mediated by Cx31.3 were recorded by using Orbit mini planar bilayer system (Nanion Technologies). Lipid bilayer was formed with a 3:1 mixture of POPE and POPG, 10 mg/ml solubilized in nonane. The proteoliposomes were added to the cis side of the bilayer, and channel incorporation was monitored at the holding potential of −50 mV. Recordings were made with a symmetrical 300 mM KCl, 10-fold KCl gradient (cis, 30 mM KCl//trans, 300 mM KCl), bi-ionic anion conditions (cis, 10 mM KCl and 290 mM K-isethionate//trans, 290 mM KCl and 10 mM K-isethionate), or bi-ionic cation conditions (cis, 300 mM NMDG-Cl//trans, 300 mM KCl) at pH 7.5 buffered with 20 mM Hepes. K-isethionate stock solution (1.5 M) was prepared from an isethionic acid (Wako Pure Chemical Industries) titrated with KOH, and 1 M NMDG-Cl stock solution was produced from NMDG (Sigma-Aldrich) by titrating with HCl. Families of 500-ms voltage pulses from −100 to +100 mV in 10-mV increments were applied from a holding potential of zero voltage. Current signals were low-pass filtered at 625 Hz and digitized at 1.25 kHz. The relative permeability was calculated by the GHK equation with modifications as follows

$$V_{\text{rev}}=\frac{\mathrm{RT}}{F}\text{ln}[\frac{P_{K}{\left[K^{+}\right]}_{o}+P_{\text{Cl}}{\left[{\text{Cl}}^{-}\right]}_{i}+P_{\text{ise}}{\left[{\text{isethionate}}^{-}\right]}_{i}+P_{\text{NMDG}}{\left[{\text{NMDG}}^{+}\right]}_{o}}{P_{K}{\left[K^{+}\right]}_{i}+P_{\text{Cl}}{\left[{\text{Cl}}^{-}\right]}_{o}+P_{\text{ise}}{\left[{\text{isethionate}}^{-}\right]}_{o}+P_{\text{NMDG}}{\left[{\text{NMDG}}^{+}\right]}_{i}}]$$

Here, *V*_rev_ is the reversal potential (in millivolt), *R* is the gas constant (8.314 J · K^−1^ · mol^−1^), *T* is the temperature in kelvin, and *F* is the Faraday’s constant (96485 C·mol^−1^). *P_x_* represents the relative permeability for ion X. [X]_o_ and [X]_i_ are the concentration of ion X in the outside (trans side) and inside (cis side), respectively.

### ATP transport assay

The phospholipid-detergent solution at 5 mg/ml was prepared by solubilizing 40 mg of POPC (Avanti Polar Lipids) and 10 mg of POPG (Avanti Polar Lipids) in 10 ml of 20 mM *n*-dodecyl-β-d-maltoside (Anatrace) solution. Purified wild-type or R15G-mutant Cx31.3 hemichannels (15 μg) in buffer B were mixed with 0.6 ml of the phospholipid-detergent solution, and buffer B was added up to 1.5 ml of the total sample volume. The mixture was dialyzed using Spectra/Por 6000 to 8000 molecular mass cutoff membrane (Spectrum Laboratories) against 5 liters of the reconstitution buffer [20 mM Hepes (pH 7.5), 250 mM NaCl, and 2 mM β-mercaptoethanol] at 4°C for 36 hours with two buffer changes. After dialysis, the remaining detergents were further removed using Bio-Beads SM-2 resin (Bio-Rad) for 4 hours. The proteoliposomes were loaded with 10 mM ATP during four freeze-thaw cycles. To remove extraliposomal ATPs, the proteoliposome sample (400 μl) was loaded on a desalting column (PD-10 column, GE Healthcare) equilibrated with the reconstitution buffer. Protein-free liposomes were also prepared by the same protocol except the protein-adding step. The amount of proteoliposomes/liposomes eluted from the desalting column was monitored by measuring the ultraviolet absorbance at 260 and 280 nm. To determine the number of ATPs in the eluted sample, 10 μl of the sample was mixed with 90 μl of the luciferase reaction mixture following the manufacturer’s instruction (ATP Determination Kit, Invitrogen). The reaction mixture additionally included 200 mM NaCl to minimize the osmotic pressure between the inside and outside of liposomes. Luminescence was immediately measured on a microplate reader (Synergy HT, BioTek), and the ATP count was estimated from the standard curve that we produced under the same buffer conditions. ATPs outside liposomes were measured immediately after gently mixing the luciferase reaction mixture and the liposome sample. To measure the total ATPs in the sample, liposomes were treated with Triton X-100 at 0.2% for 30 min. The number of ATPs retained inside liposomes was determined by subtracting the intraliposomal ATP count from the total ATP count.

## Supplementary Material

aba4996_SM.pdf

## SUPPLEMENTARY MATERIALS

Supplementary material for this article is available at http://advances.sciencemag.org/cgi/content/full/6/35/eaba4996/DC1

## Appendix

View/request a protocol for this paper from *Bio-protocol*.
