# Supplementary material for: Cryo-EM structure of human Cx31.3/GJC3 connexin hemichannel
Source: Sci Adv. 2020 Aug 28;6(35):eaba4996. doi: 10.1126/sciadv.aba4996 (PMC7455182; doi:10.1126/sciadv.aba4996)
Supplement: aba4996_SM.pdf [file aba4996_SM.pdf]

Science Advances
AAAS

advances.sciencemag.org/cgi/content/full/6/35/eaba4996/DC1

Supplementary Materials for

Cryo-EM structure of human Cx31.3/GJC3 connexin hemichannel

Hyuk-Joon Lee, Hyeongseop Jeong, Jaekyung Hyun, Bumhan Ryu, Kunwoong Park, Hyun-Ho Lim, Jejoong Yoo, Jae-Sung Woo*

*Corresponding author. Email: jaesungwoo@korea.ac.kr

Published 28 August 2020, *Sci. Adv.* **6**, eaba4996 (2020)
DOI: 10.1126/sciadv.aba4996

This PDF file includes:

Figs. S1 to S10
Table S1

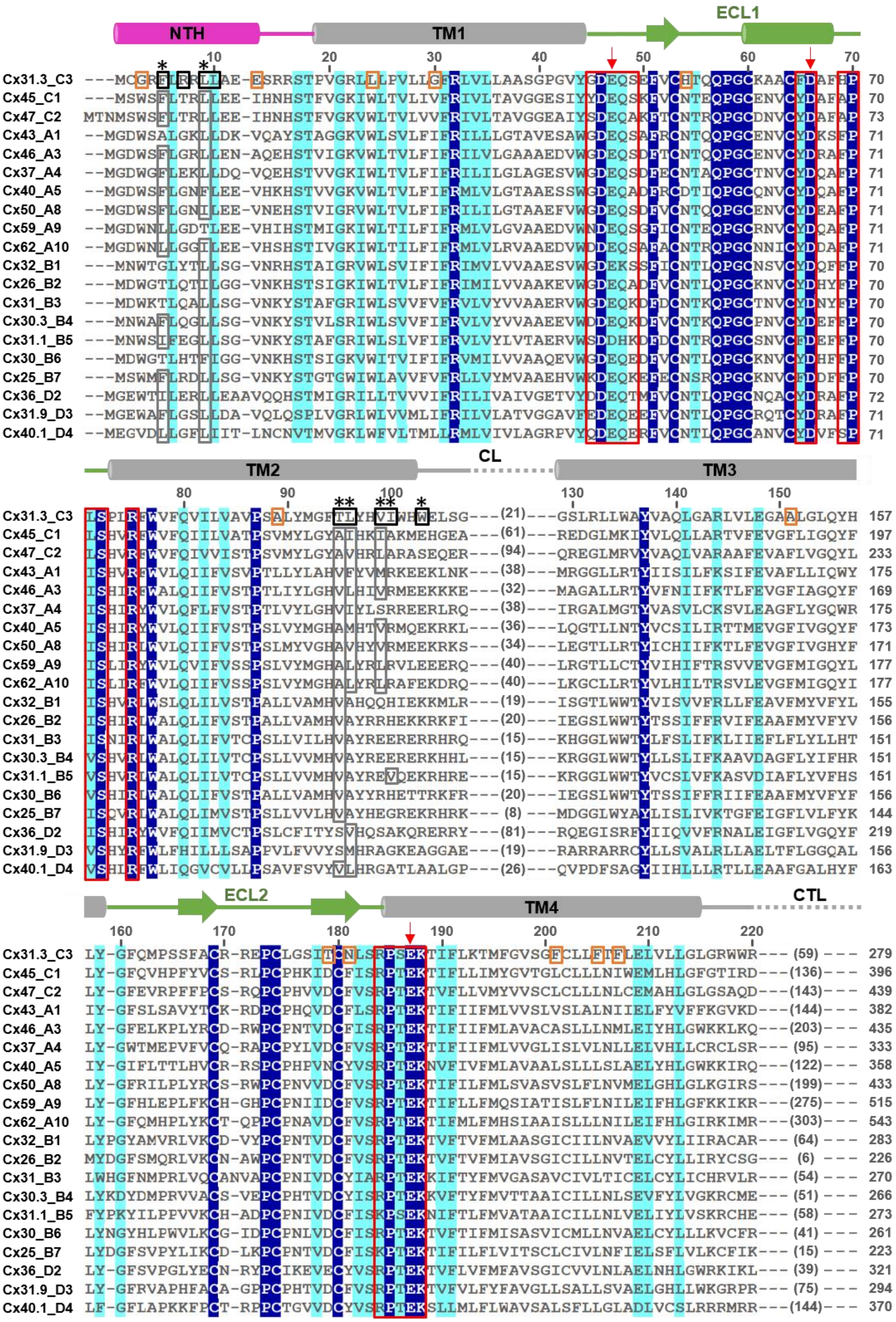

Fig. S1. – continues on next page

Fig. S1. Multiple sequence alignment of 20 human connexin family proteins.
The sequence alignment does not contain a highly diversified member Cx23. Blue shades indicate 100% identically conserved residues. Cyan shades indicate 90% identically conserved or 100% similar residues. Orange boxes indicate the Cx31.3-specific residues to which the corresponding residues are highly conserved in other human connexin proteins. Black boxes indicate the residues involved in the NTH-TM2 interaction. The residues participating in the intramolecular NTH-TM2 interaction are highlighted by asterisks, and their corresponding residues similarly conserved in other connexins are indicated by gray boxes. Red boxes indicate the residues lining the Ca^{2+}-binding tunnel. Red arrows indicate three acidic residues in the Ca^{2+}-binding tunnel. The secondary structures (cylinders, α-helices; arrows, β-strands) from the Cx31.3 hemichannel structure are shown on the top of the sequence alignment. NTH, TM helices, and ECLs are colored in magenta, gray, and green, respectively. Flexible regions with low sequence homology within the cytoplasmic loop (CL) and C-terminal loop (CTL) are not shown, and the numbers of omitted amino acids are shown in parentheses.

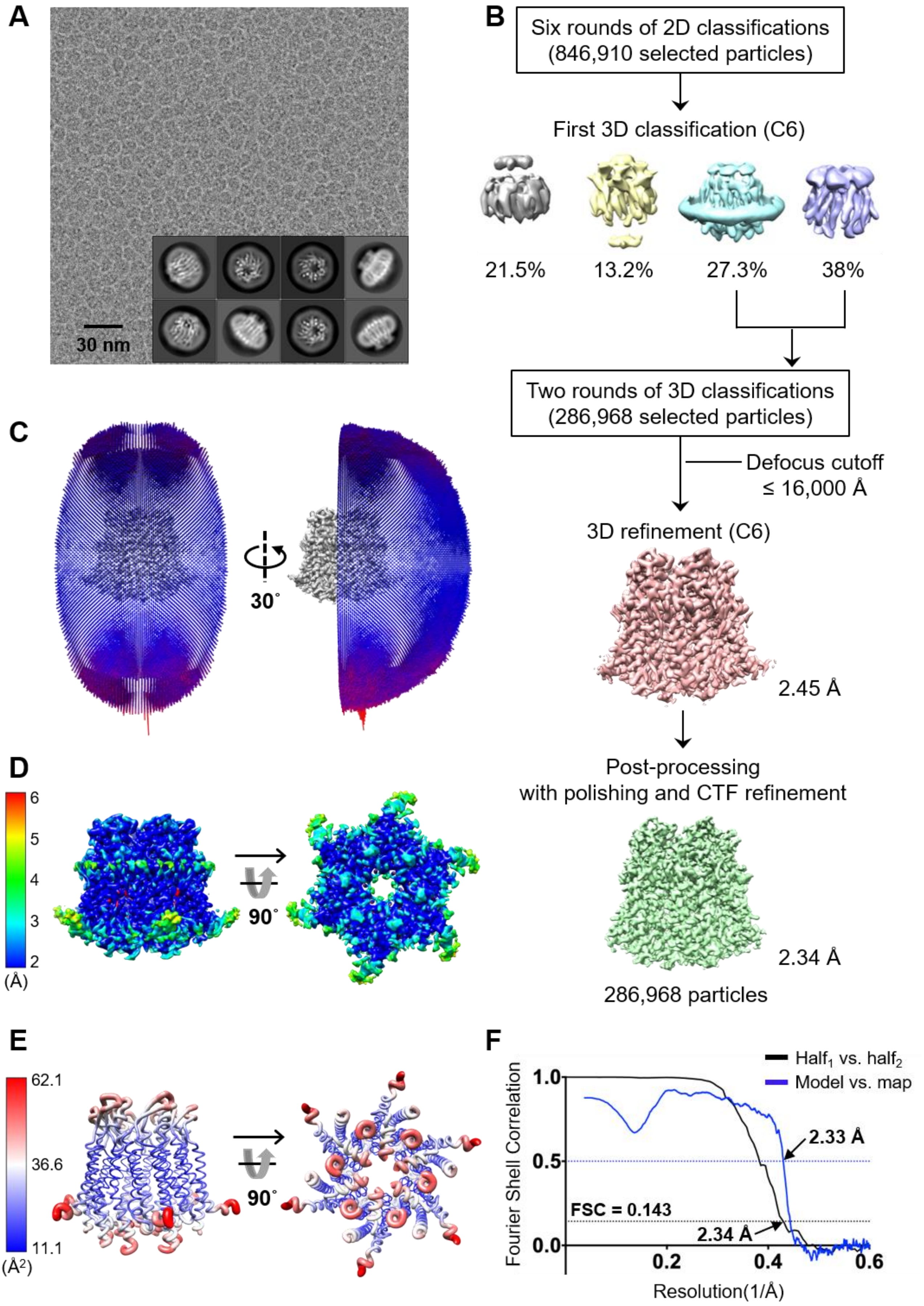

Fig. S2. – continues on next page

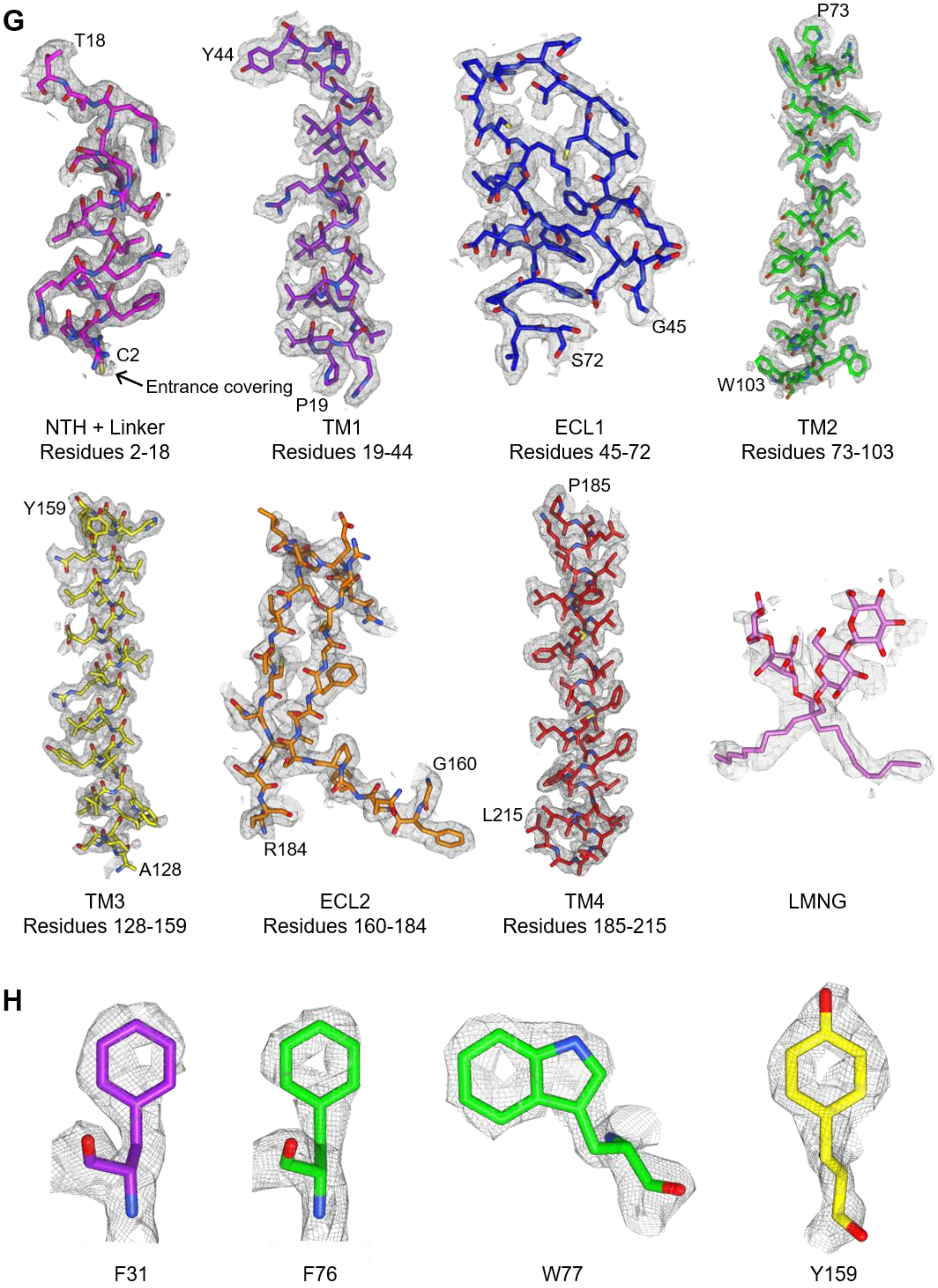

Fig. S2. – continues on next page

Fig. S2. Cryo-EM image processing and local cryo-EM density maps of Cx31.3 hemichannel in the absence of calcium ions.

(A) A representative cryo-electron micrograph and 2D class averages (inset) of Cx31.3 hemichannel in the absence of calcium ions. A 30 nm scale bar is shown in the micrograph.

(B) A flow chart that describes cryo-EM image processing steps (see Materials and Methods).

(C) Angular distributions of all particles used in the final 3D reconstruction with C6 symmetry imposition.

(D) The cryo-EM map colored according to local resolution estimated using ResMap. The local resolution gradient ranges from 2 Å (blue) to 6 Å (red).

(E) B-factor putty representation of the refined atomic model. The putty thickness and colour represent the B-factor of each residue ranging from 11.1 $Å^2$ (blue) to 62.1 $Å^2$ (red).

(F) Fourier shell correlations between the unfiltered cryo-EM half maps (black line) and between the full map and refined atomic model (blue line). The former indicates the estimated resolution of 2.34 Å at 0.143 cutoff and the latter indicates the estimated resolution of 2.33 Å at 0.5 cutoff.

(G) The Cx31.3 structure is separated into seven regions, presented in sticks with the corresponding map densities, and colored as in Figure 1D. The first and last residues in the structures are labeled. LMNG model bound to the outer surface of TMDs is also shown with the corresponding map density.

(H) Representative high resolution features of EM density map as shown by clear aromatic rings. The fitted atomic models are colored as in (G).

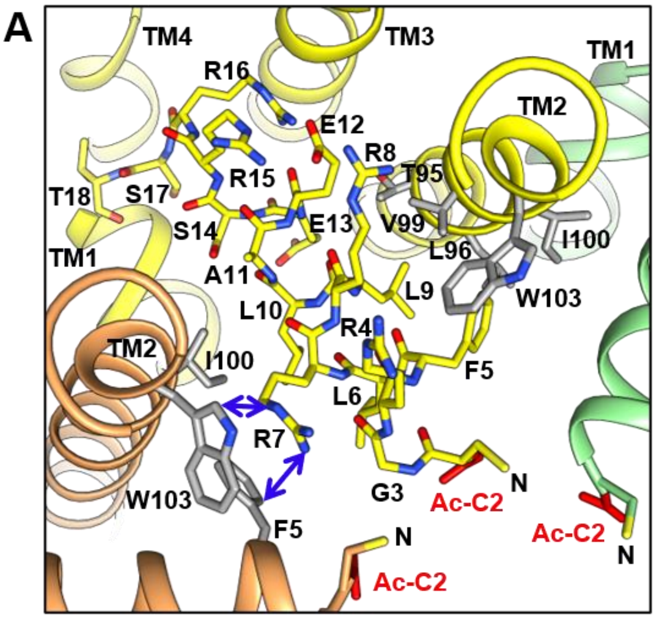

B

Residues	Sequence	Intensity ($\times 10^6$)
1 - 13	MCGRFLRRLLAEE	Not detected
2 - 13	CGRFLRRLLAEE	54.767
2 - 13	Ac-CGRFLRRLLAEE	20.474
112 - 120	EETLIQGRE	26.47
113 - 120	ETLIQGRE	2693.8
114 - 120	TLIQGRE	42050
237 - 252	STRRHKKATDSLPVVE	81591
253 - 259	TKEQFQE	5414.3
253 - 269	TKEQFQEAVPGRSLAQE	835.42
256 - 269	QFQEAVPGRSLAQE	324.72
260 - 269	AVPGRSLAQE	158620
270 - 279	KQRPVGPRDA	883.17

* Ac : N-terminal acetylation

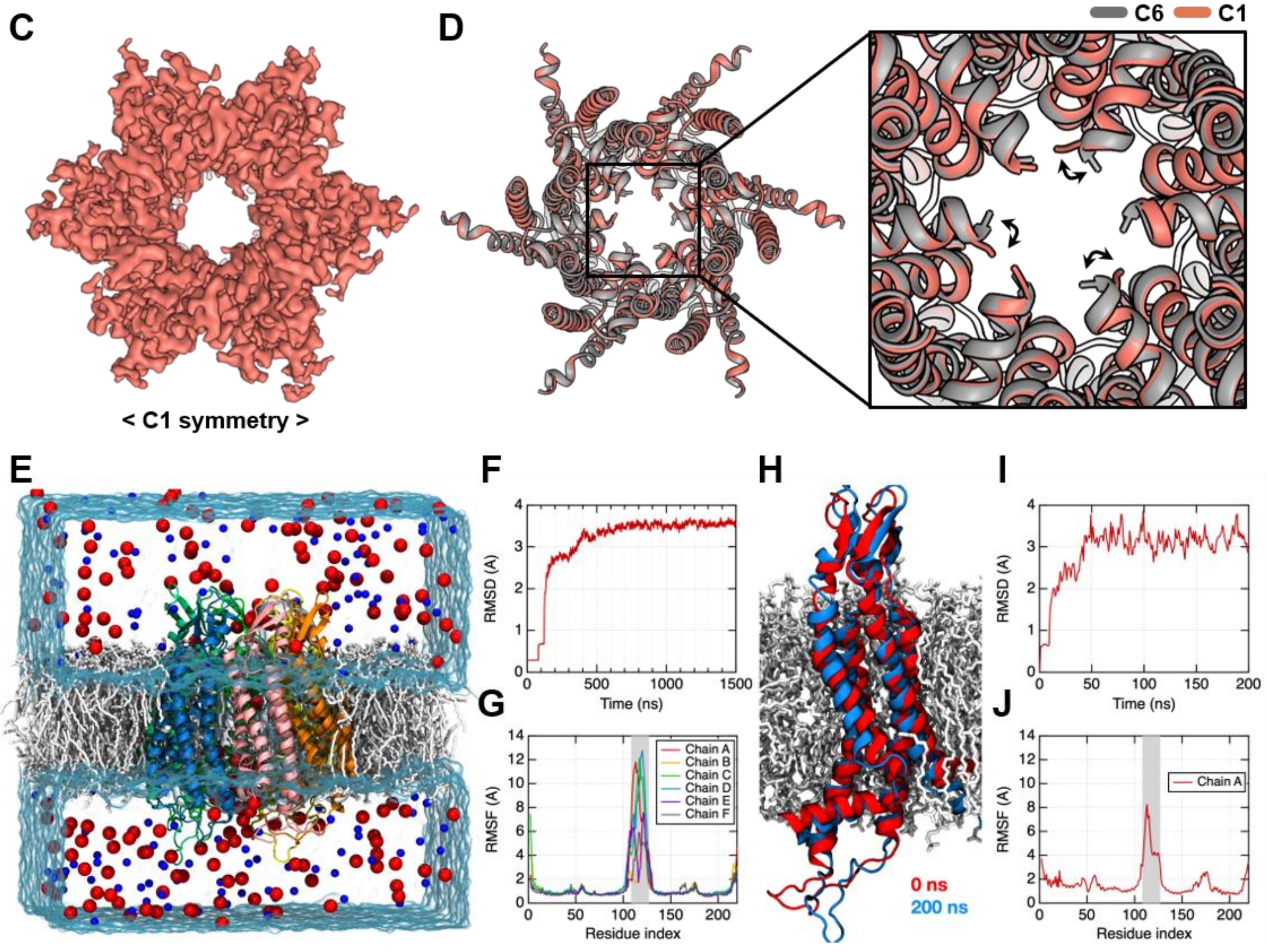

Fig. S3. – **continues on next page**

Fig. S3. The conformational stability and N-terminal modification of Cx31.3 hemichannel.

(A) Detailed view of the NTH-TM2 interaction. NTH and the following loop are shown in yellow sticks and side chains of the TM2 residues interacting with NTH are also shown in gray sticks. Blue arrows indicate cation-π interactions. The acetylation of C2 is modelled and shown in red sticks.

(B) Mass spectrometry data of purified Cx31.3. The first methionine residue was not detected in any N-terminal peptides. Acetylation of C2 (Ac-C) is highlighted by red letters.

(C) Cytoplasmic view of Cx31.3 hemichannel structure calculated with C1 (red) symmetry.

(D) Superposition of two structures with C1 (red) and C6 (gray) symmetries showing the conformational differences in three N-termini (black arrows).

(E) Molecular dynamics simulation setup of Cx31.3 hemichannel (cartoon representation in the center) embedded in the lipid bilayer of a 4:1 mixture of POPC and POPE (white molecular representations in a cutaway view). Blue semi-transparent surface indicates the aqueous solution containing 150 mM K^+ (blue spheres) and Cl^- (red spheres) ions.

(F) Root mean square deviation (RMSD) of Cx31.3 hemichannel as a function of time. Unstructured CL loops (residue index 108 to 126) were excluded in the computation of RMSD. Note that the channel was restrained during the first 130 ns.

(G) Root mean square fluctuation (RMSF) of each chain averaged over the last one-microsecond trajectory. Gray background indicates the unstructured loop region.

(H) Comparison of the Cx31.3 protomer structure from the Cryo-EM experiment (red) with the structure after 200-ns simulation (blue).

(I) RMSD of the protomer as a function of time, excluding the unstructured loop.

(J) RMSF as a function of residue index averaged over the last 150-ns trajectory.

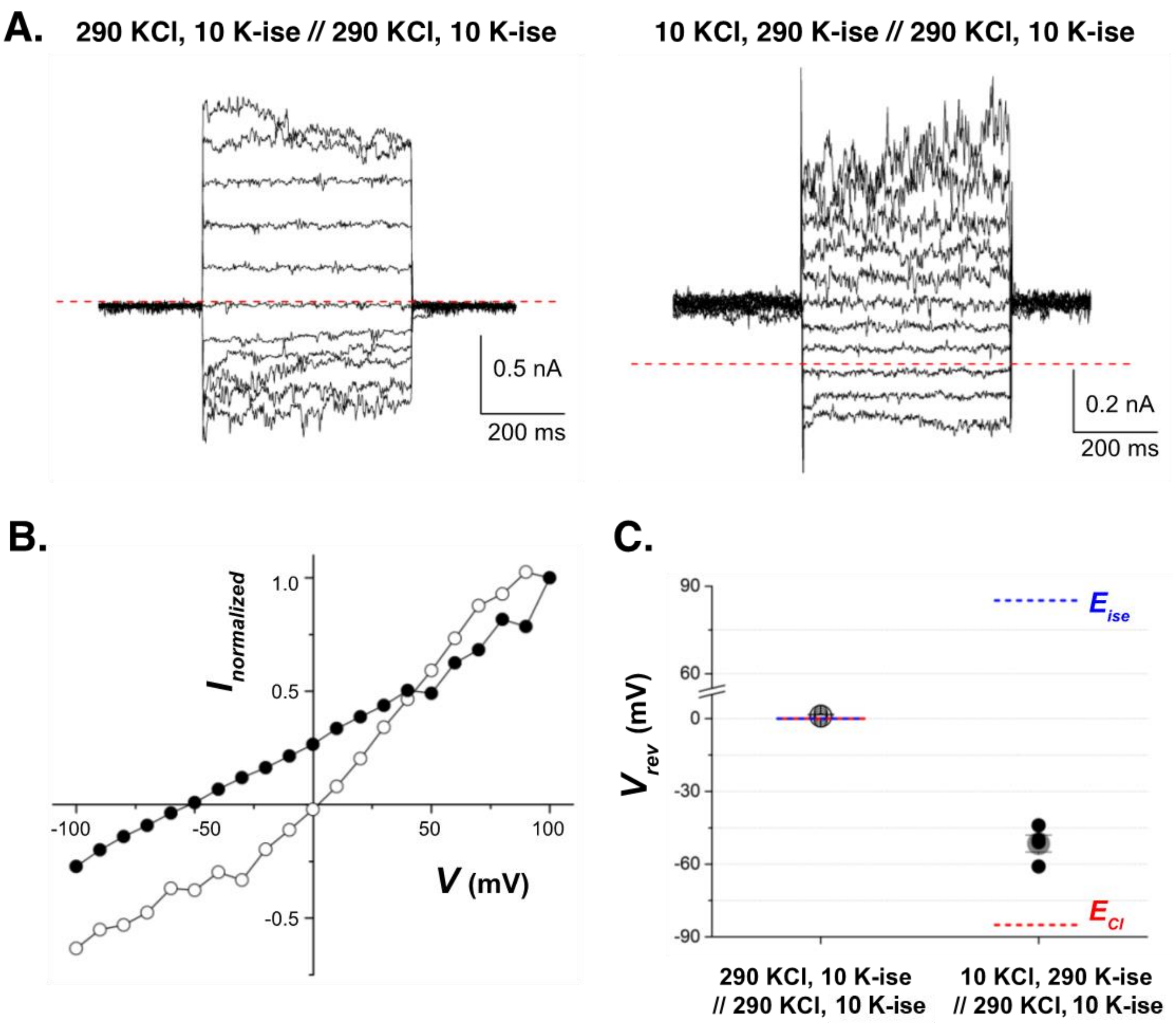

Fig. S4. The permeability of a polyatomic anion through the Cx31.3 hemichannels.
(A) Representative current traces of Cx31.3 hemichannels in the presence of polyatomic anion, isethionate. Ion gradients across the bilayer are indicated (in mM concentration, *cis*-side // *trans*-side). Ionic currents were recorded under a symmetrical KCl and K-isethionate condition (left) and an asymmetrical anion condition (right). Ionic currents were evoked by 500-ms test pulses from -100 mV to +100 mV with 10-mV increments. For clarity, currents are displayed in 20-mV increments. Red dashed lines mark zero-current level.
(B) I-V curves in a symmetrical KCl and K-isethionate condition (empty circle) and an asymmetrical anion condition (filled circle). Ionic currents averaged between 290 and 490 ms into the test pulses are normalized to the values at +100 mV.
(C) Reversal potentials (V_{rev}) were determined by null-point measurement in a symmetrical KCl and K-isethionate condition (empty circle, n=4) and an asymmetrical anion condition (filled circle, n=4). The Nernst potentials for the Cl^- (red dashed lines) and isethionate (blue dashed line) are displayed in the indicated gradient. Each bar represents the mean ± SEM.

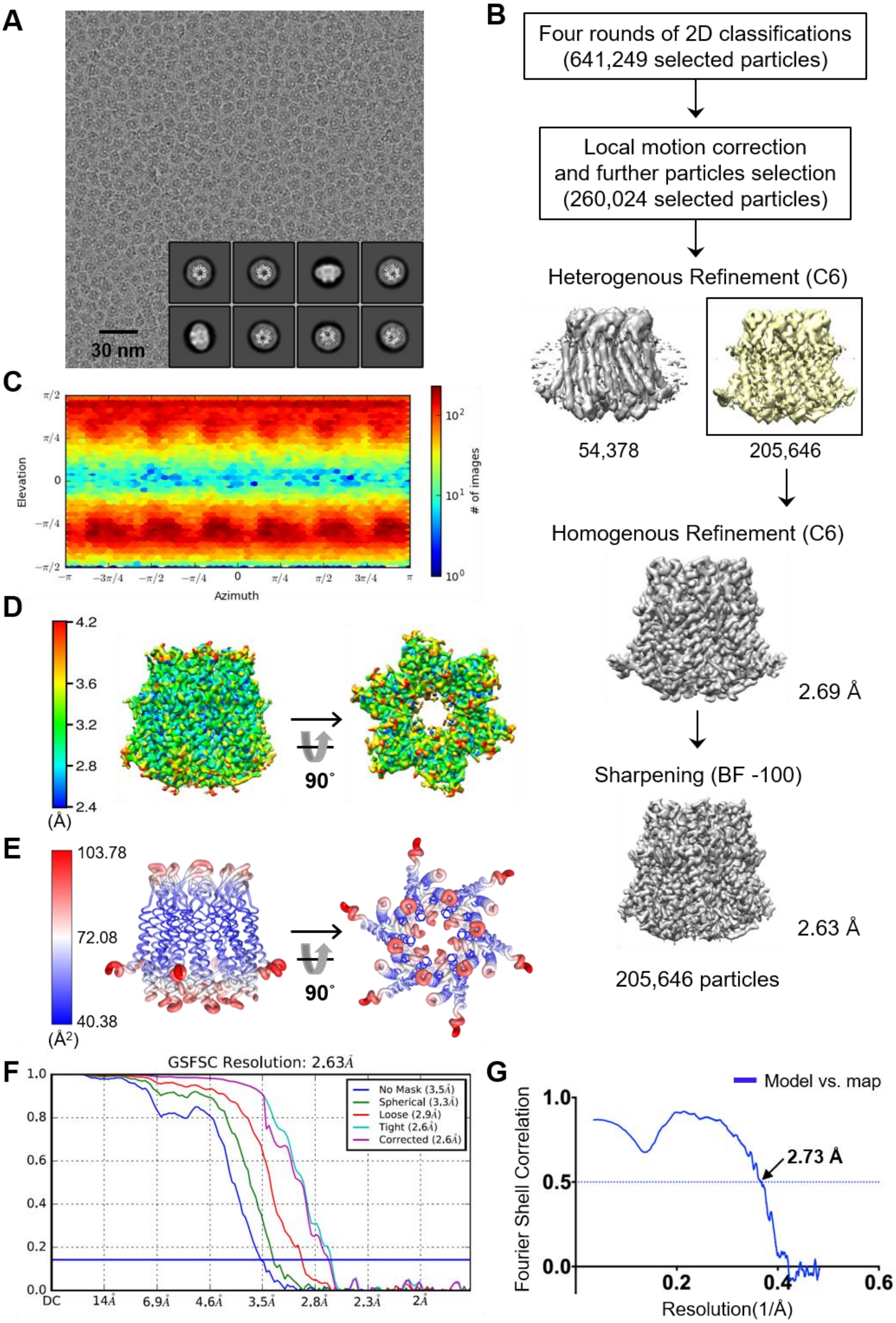

Fig. S5. – continues on next page

Fig. S5. Cryo-EM image processing of the R15G mutant hemichannel of Cx31.3.
(A) A representative cryo-electron micrograph and 2D class averages (inset) of Cx31.3 R15G mutant hemichannel. A 30 nm scale bar is shown in the micrograph.
(B) A flow chart that describes cryo-EM image processing steps (see Materials and Methods).
(C) 2D plot illustrating angular orientation distributions of all particles used in the final 3D reconstruction.
(D) The cryo-EM map colored according to local resolution estimated using ResMap. The local resolution gradient ranges from 2.4 Å (blue) to 4.2 Å (red).
(E) B-factor putty representation of the refined atomic model. The putty thickness and colour represent the B-factor of each residue ranging from 40.38 $Å^2$ (blue) to 103.78 $Å^2$ (red).
(F) Fourier shell correlation between the unfiltered cryo-EM half maps with various masking schemes indicates the estimated resolution of 2.6 Å at 0.143 cutoff.
(G) Fourier shell correlation between the cryo-EM full map and the refined atomic model (blue) indicates the estimated resolution of 2.73 Å at 0.5 cutoff.

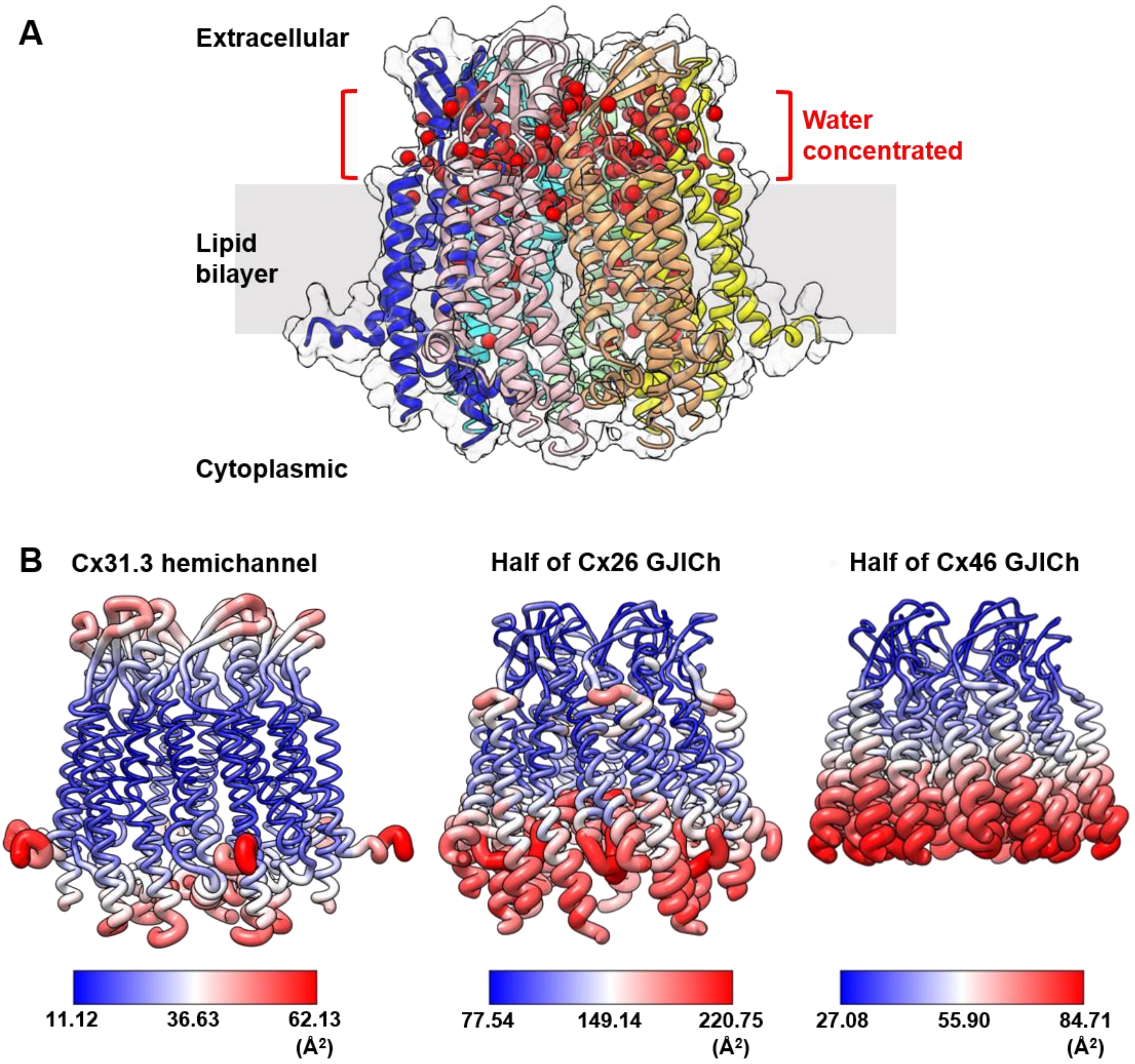

Fig. S6. Strong interaction of ECLs with water molecules and relatively high flexibility of ECLs.

(A) 204 identified water molecules are shown in red spheres.

(B) Comparison of B-factor distribution between Cx31.3 hemichannel, half of Cx26 GJICh (PDB ID: 2ZW3), and half of Cx46 GJICh (PDB ID: 6MHQ). ECLs in Cx31.3 hemichannel show relatively higher B-factors than TM helices, while ECLs in Cx26 and Cx46 GJIChs are the regions with the lowest B-factors.

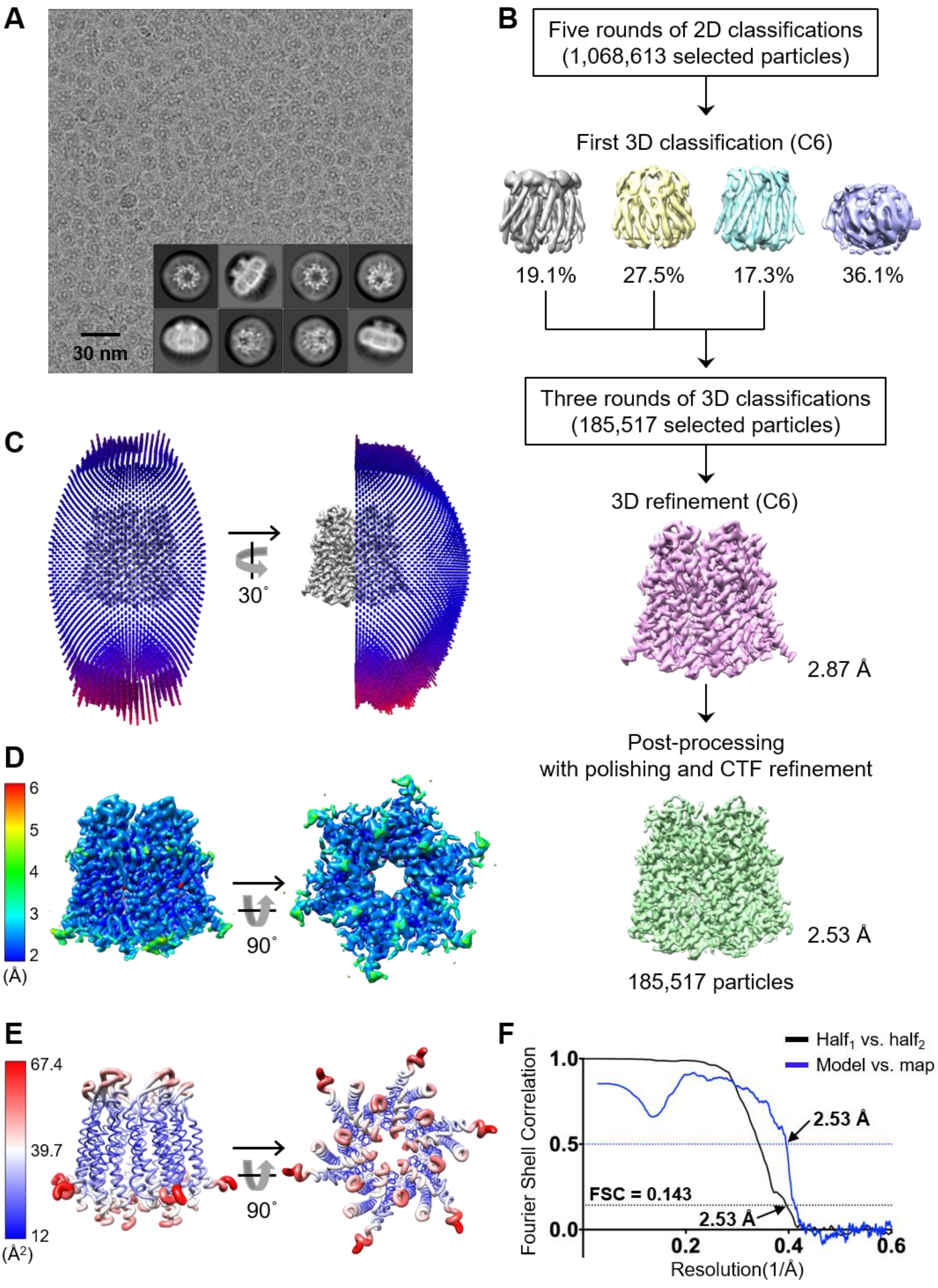

Fig. S7. – continues on next page

Fig. S7. Cryo-EM image processing of Cx31.3 hemichannel in the presence of calcium ions.

(A) A representative cryo-electron micrograph and 2D class averages (inset) of Cx31.3 hemichannel in the presence of calcium ions. A 30 nm scale bar is shown in the micrograph.
(B) A flow chart that describes cryo-EM image processing steps (see Materials and Methods).
(C) Angular distribution of all particles used in the final 3D reconstruction with C6 symmetry imposition.
(D) The cryo-EM map colored according to local resolution estimated using ResMap. The local resolution gradient ranges from 2 Å (blue) to 6 Å (red).
(E) B-factor putty representation of the refined atomic model. The putty thickness and colour represent the B-factor of each residue ranging from 12 $Å^2$ (blue) to 67.4 $Å^2$ (red).
(F) Fourier shell correlations between the unfiltered cryo-EM half maps (black line) and between the full map and refined atomic model (blue line). The former indicates the estimated resolution of 2.53 Å at 0.143 cutoff and the latter indicates the estimated resolution of 2.53 Å at 0.5 cutoff.

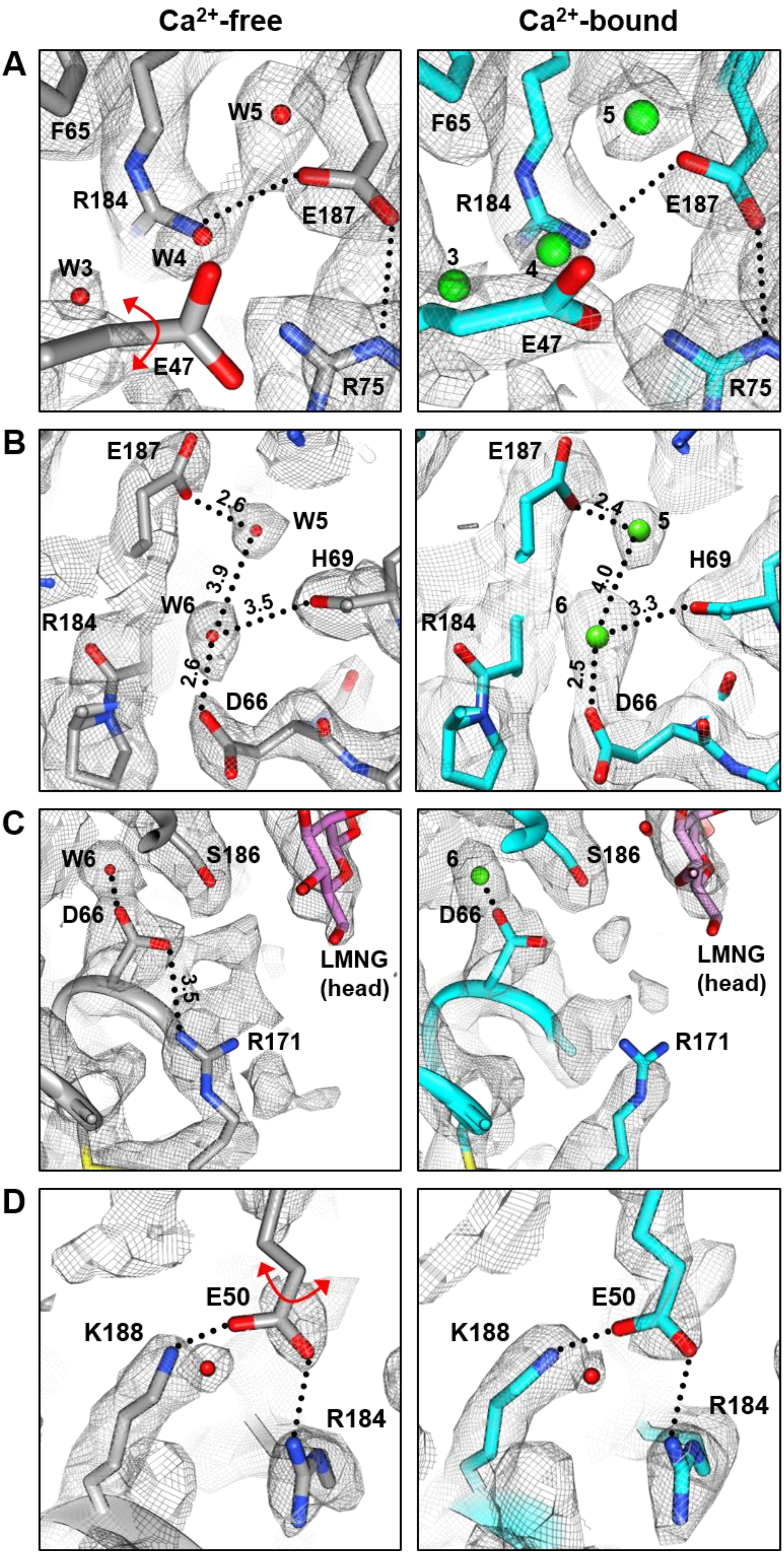

Fig. S8. – continues on next page

Fig. S8. Detailed structural changes in and around the Ca^{2+}-binding tunnel of Cx31.3 hemichannel by calcium ions.

(A) The map density of E47 is much stronger in the Ca^{2+}-bound state (right) than in the Ca^{2+}-free state (left). Map densities in both panels are shown at the same contour level ($\sigma = 5.0$). A red two-way arrow indicates high flexibility of the side chain. The dotted lines indicate salt bridges.

(B) The map density at the W6 position is significantly stronger in the Ca^{2+}-bound state (right) than in the Ca^{2+}-free state (left). Map densities in both panels are shown at the different contour level (Ca^{2+}-free, $\sigma = 6.9$ and Ca^{2+}-bound, $\sigma = 5.3$). It should be noted that, in contrast with the W6 density, the map densities for the residues around W6 are slightly stronger in the Ca^{2+}-free state than the Ca^{2+}-bound state. In addition, the side chain conformation of D66 also slightly changed resulting in a shorter distance to putative Ca^{2+} position 6 (2.5 Å) than the distance to W6 (2.6 Å). Ca^{2+}-coordinating interactions are indicated by dotted lines.

(C) The map density of R171 has shifted far from D66 in the Ca^{2+}-bound state (right), compared with the Ca^{2+}-free state (left). Map densities in both panels are shown at the same contour level ($\sigma = 4.3$). The dotted lines indicate salt bridges.

(D) The map density of E50 is significantly stronger in the Ca^{2+}-bound state (right) than in the Ca^{2+}-free state (left). For a fair comparison, we applied different contour levels to the two structures (Ca^{2+}-free, $\sigma = 7.4$ and Ca^{2+}-bound, $\sigma = 7.9$) where the residues around E50 have similar map sizes. The dotted lines indicate salt bridges, and a red two-way arrow indicates flexibility of the side chain.

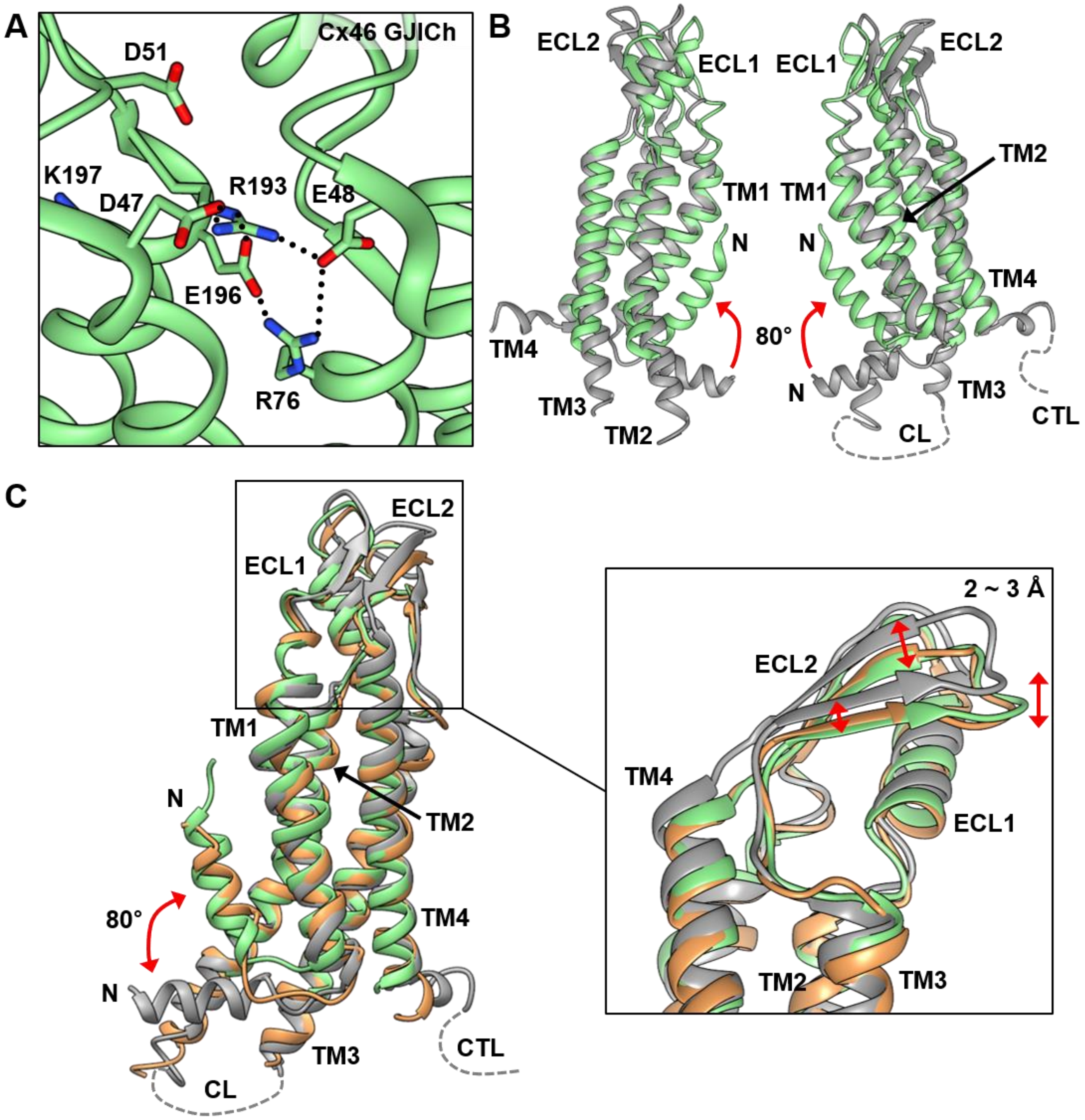

Fig. S9. – continues on next page

Fig. S9. Detailed structural comparison of Cx31.3 hemichannel with Cx46 GJICh.

(A) Salt-bridge networks in and around Ca^{2+}-binding tunnels of Cx46 (PDB ID: 6MHQ) GJICh. The residues involved in both circular and linear salt-bridge networks are shown in sticks and labeled (See Figure 6B). Salt bridges with distances less than 4 Å are indicated by black dotted lines.

(B) Structural differences in ECLs and TMDs between Cx31.3 hemichannel (gray) and Cx46 GJICh (green). The overall structures of Cx31.3 and Cx46 channels were aligned, and two interfacing connexins in each channel are shown. ECLs and TM helices of Cx46 GJICh show slight rotation and tilting towards the pore compared to Cx31.3 hemichannel. Red arrows indicate significant conformational shifts of NTHs in Cx46 GJICh compared to Cx31.3 hemichannel.

(C) Structural alignment of Cx31.3, Cx26 (PDB ID: 2ZW3), and Cx46 protomers. TM helices are well superposed. However, the close-up view in the box shows that the significant conformational difference in ECLs still exists in the protomer level.

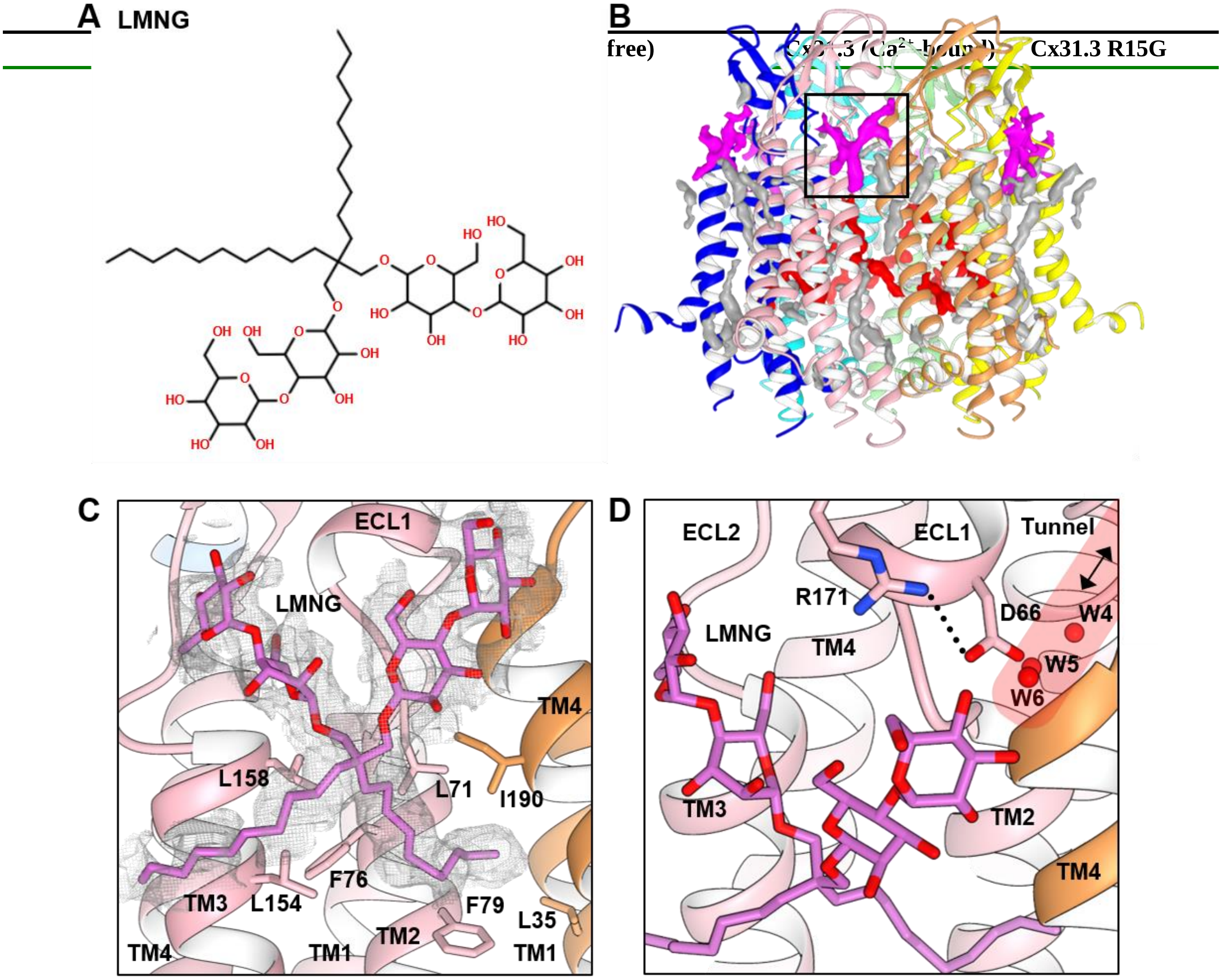

Fig. S10. The identification of bound LMNG molecules.

(A) The chemical structure of LMNG.

(B) Strong density maps surrounding the Cx31.3 hemichannel structure are manually selected and shown as pink, gray, and red surfaces. Pink densities in the black box correspond to LMNG molecules.

(C) Detailed view of the LMNG binding to the outer surface of the hemichannel. LMNG is shown in pink sticks. The density map for LMNG is shown in gray mesh. Hydrophobic residues mainly involved in the interaction with LMNG are shown in sticks and labeled.

(D) The head group of LMNG is located close to R171. The salt bridge between D66 and R171 is indicated by a dotted line. The Ca^{2+}-binding tunnel is indicated by an arrow, and water molecules in the tunnel are shown as red spheres.

	C6 symmetry<br>EMD-0825<br>PDB 6L3T	C1 symmetry	EMD-0826<br>PDB 6L3U	EMD-0827<br>PDB 6L3V
**Data collection and processing**				
Microscope	Titan Krios	Titan Krios	Titan Krios	Titan Krios
Camera	Falcon 3EC	Falcon 3EC	Falcon 3EC	Falcon 3EC
Magnification	96,000	96,000	96,000	75,000
Voltage (kV)	300	300	300	300
Electron exposure (e^{-}/Å^2)	29.8	29.8	39.9	44.9
Defocus range (μm)	–0.8 ~ –2.7	–0.8 ~ –2.7	–0.8 ~ –2.7	–0.8 ~ –2.7
Pixel size (Å)	0.673	0.673	0.673	0.863
Software	RELION 3.0	RELION 3.0	RELION 3.0	cryoSPARC v2
Symmetry imposed	C6	C1	C6	C6
Initial particle images (no.)	1,479,056	1,479,056	2,855,688	1,834,380
Final particle images (no.)	286,968	286,968	185,517	205,646
Overall map resolution (Å)				
FSC threshold 0.143	2.34	2.62	2.53	2.63
**Refinement**				
Software	Phenix 1.17 real-space-refine	Phenix 1.17 real-space-refine	Phenix 1.17 real-space-refine	Phenix 1.17 real-space-refine
Initial model used (PDB code)	N/A	6L3T	6L3T	6L3T
Model resolution (Å)				
FSC threshold 0.5	2.3	2.6	2.5	2.7
Map sharpening *B* factor (Å^2)	–67.7	–59.4	–80.6	–100
Model composition				
Non-hydrogen atoms	10,134	9,516	10,110	9,888
Protein residues	1,206	1,206	1,206	1,206
Ligands	LMN : 6	N/A	LMN : 6<br>Ca^{2+}-ion : 6	LMN : 6
B factors (Å^2)				
Protein	27.43	40.81	30.14	61.52
Ligand	26.86	N/A	27.41	59.60
R.m.s. deviations				
Bond lengths (Å)	0.006	0.006	0.006	0.004
Bond angles (°)	0.952	0.808	0.840	0.8
Validation				
MolProbity score	1.26	1.11	1.09	1.37
Clashscore	1.20	1.36	0.7	1.05
Poor rotamers (%)	3.61	2.11	3.01	5.45
Ramachandran plot				
Favored (%)	98.98	98.82	98.05	99.49
Allowed (%)	1.02	1.18	1.95	0.51
Disallowed (%)	0	0	0	0

Table S1. Cryo-EM data collection, refinement, and validation statistics.
